# Integrating machine learning and single-cell sequencing to identify shared biomarkers in type 1 diabetes mellitus and clear cell renal cell carcinoma

**DOI:** 10.3389/fonc.2025.1543806

**Published:** 2025-03-03

**Authors:** Yi Li, Rui Zeng, Yuhua Huang, Yumin Zhuo, Jun Huang

**Affiliations:** ^1^ Department of Ultrasound, The Affiliated Guangdong Second Provincial General Hospital of Jinan University, Guangzhou, China; ^2^ Department of Pathology, School of Medicine, South China University of Technology, Guangzhou, China; ^3^ Department of Pathology, Guangdong Provincial People’s Hospital (Guangdong Academy of Medical Sciences), Guangzhou, China; ^4^ Department of Ultrasound, The First Affiliated Hospital of Jinan University, Guangzhou, China; ^5^ Department of Urology, The First Affiliated Hospital of Jinan University, Guangzhou, China

**Keywords:** type 1 diabetes mellitus, clear cell renal cell carcinoma, key genes, machine learning, single cell sequencing

## Abstract

**Purpose:**

Type 1 diabetes mellitus (T1DM), as an autoimmune disease, can increase susceptibility to clear cell renal cell carcinoma (ccRCC) due to its proinflammatory effects. ccRCC is characterized by its subtle onset and unfavorable prognosis. Thus, the aim of this study was to highlight prevention and early detection opportunities in high-risk populations by identifying common biomarkers for T1DM and ccRCC.

**Methods:**

Based on multiple publicly available datasets, WGCNA was applied to identify gene modules closely associated with T1DM, which were then integrated with prognostic DEGs in ccRCC. Subsequently, the LASSO and SVM algorithms were employed to identify shared hub genes between the two diseases. Additionally, clinical samples were used to validate the expression patterns of these hub genes, and scRNA-seq data were utilized to analyze the cell types expressing these genes and to explore potential mechanisms of cell communication.

**Results:**

Overall, three hub genes (KIF21A, PIGH, and RPS6KA2) were identified as shared biomarkers for TIDM and ccRCC. Analysis of clinical samples and multiple datasets revealed that KIF21A and PIGH were significantly downregulated and that PIG was upregulated in the disease group. KIF21A and PIGH are mainly expressed in NK and T cells, PRS6KA2 is mainly expressed in endothelial and epithelial cells, and the MIF signaling pathway may be related to hub genes.

**Conclusion:**

Our results demonstrated the pivotal roles of hub genes in T1DM and ccRCC. These genes hold promise as novel biomarkers, offering potential avenues for preventive strategies and the development of new precision treatment modalities.

## 1 Introduction

Diabetes mellitus (DM) affects approximately 463 million people worldwide, with type 1 diabetes mellitus (T1DM) accounting for nearly 10%. Remarkably, the prevalence of T1DM is increasing globally (1, 2). Although there is no definitive consensus on the exact pathogenic mechanism of T1DM, it is fundamentally characterized as a chronic autoimmune disorder involving the destruction of insulin-producing pancreatic islet cells (β cells) (3). By identifying autoantibodies, autoreactive CD8+ T cells are the primary immune cells responsible for the death of β cells, and islet-specific CD4+ T cells can produce cytokines that promote the activation of B cells and islet-specific CD8+ T cells (4, 5). Additionally, recent studies have emphasized the significance of natural killer cells (NKs) in autoimmune contexts. NKs can eliminate specific cells, generate memory cells, respond to antigens, and interact with CD8+ T cells, promoting immune-mediated assaults on β cells (6, 7).

T1DM increases cancer risk, and previous research has highlighted its connection to renal cell carcinoma (RCC). Coexisting T1DM in RCC patients increases the risk of recurrence and distant metastasis, contributing to a poorer prognosis (8). Clear cell renal cell carcinoma (ccRCC) constitutes up to 80% of all pathological subtypes of RCC (9), and nearly one-third of ccRCC patients present with metastases at the initial diagnosis (10). The link between RCC and diabetes has become increasingly evident over the years, both in terms of incidence and prognosis (11, 12). DM, a major consequence of metabolic syndrome, is more prevalent in ccRCC patients, and those with DM and ccRCC tend to have worse outcomes compared to ccRCC patients without DM. Diabetes is associated with higher recurrence rates, increased distant metastases, and reduced survival rates (8). Epidemiological studies also highlight that individuals with diabetes have a significantly higher risk of RCC compared to non-diabetic individuals (13). This connection is particularly prominent in conditions associated with metabolic syndrome, such as T1DM.

The tumor microenvironment (TME) in ccRCC is characterized by a substantial presence of tumor-infiltrating immune cells (14). Although a connection between T1DM and immune cell activity exists, the exact extent of its influence on the TME still needs to be understood. Therefore, it is imperative to assess the potential impact of other related diseases on the TME. Therefore, employing machine learning techniques that integrate multiple diseases can facilitate a more comprehensive analysis of meaningful biomarkers (15, 16). The primary aim of this study was to identify common biomarkers shared between T1DM and ccRCC, with the objective of establishing a more comprehensive early diagnostic model for ccRCC based on novel potential serum biomarkers. Another objective of this study was to identify biomarkers with increased sensitivity and specificity for the early screening and diagnosis of ccRCC in T1DM patients. Additionally, we hypothesized that these identified markers may play a role in the immune mechanism underlying the pathogenesis of ccRCC. In this work, we endeavored to provide new insights into the impact of the TME.

**Figure 1:**
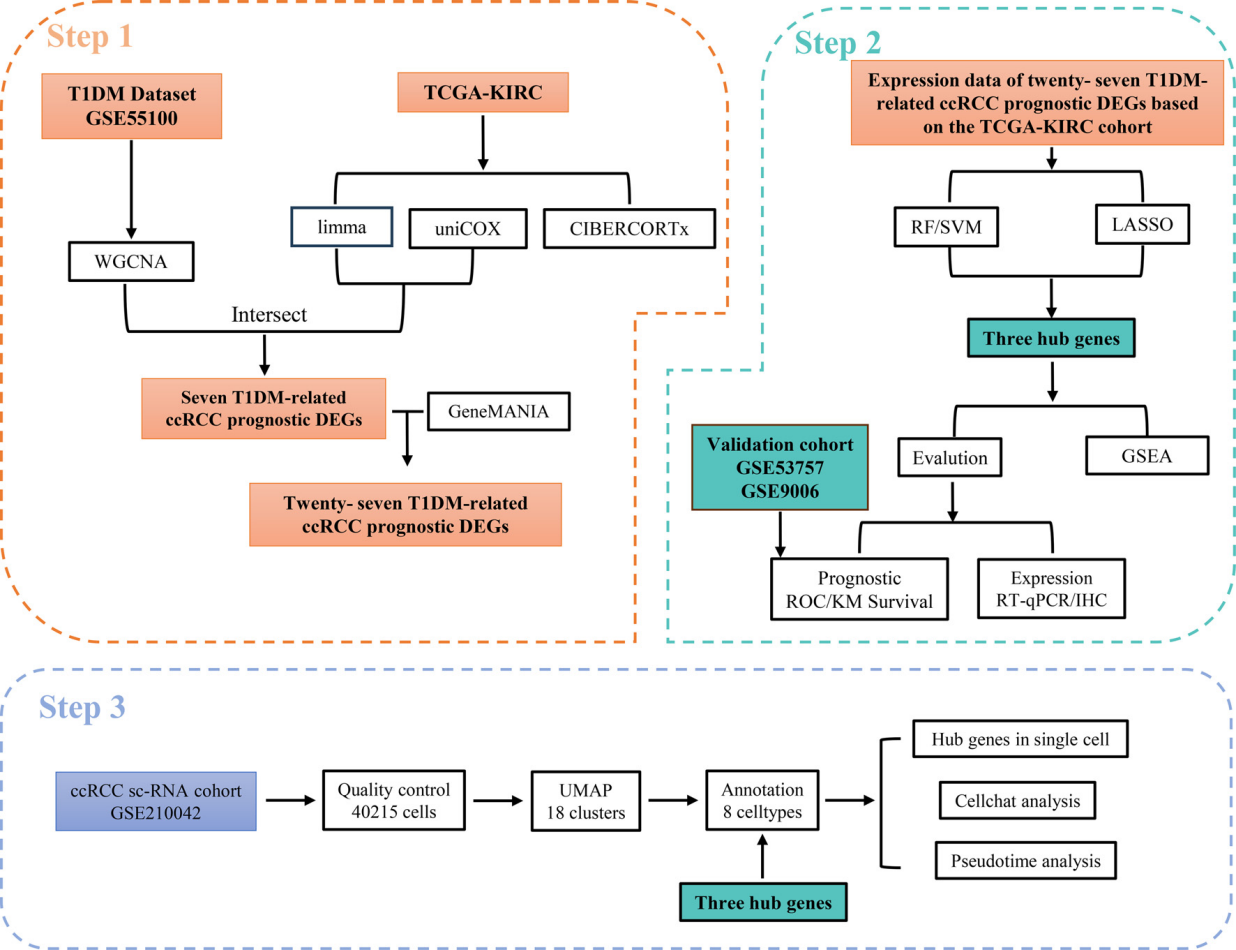
Workflow of this study. Step 1: Identified T1DM-related genes using WGCNA based on the T1DM dataset and intersected them with prognostic DEGs in ccRCC. Their associated genes were subsequently obtained using the GeneMANIA database. Step 2: Hub genes were selected from these T1DM-related ccRCC prognostic DEGs and their associated genes using machine learning algorithms. The clinical value of the hub genes was validated through ROC curve analysis in an independent validation cohort. Furthermore, the expression levels of the hub genes were confirmed via RT-qPCR and IHC, and potential shared regulatory pathways between T1DM and ccRCC were explored using GSEA. Step 3: scRNA-seq data were utilized to validate the expression of hub genes at the single-cell level and to investigate their association with immune microenvironment remodeling.

In this study, we used a variety of machine learning algorithms and weighted gene coexpression network (WGCNA) methods to screen out possible shared biomarkers between T1DM and ccRCC. We further identified the core genes closely related to the diagnosis and prognosis of T1DM patients and ccRCC patients and verified the expression levels of these genes in clinical samples. Immune infiltration analysis revealed that these core genes were closely related to the immune response of NK cells and T cells in the immune microenvironment of ccRCC, and single-cell RNA sequencing (scRNA-seq) data confirmed this result (**Figure 1**). These findings reveal potential alterations in the immune microenvironment of ccRCC in the context of T1DM and provide a new target for the prevention and treatment of ccRCC.

## 2 Materials and methods

### 2.1 Transcriptome data acquisition and processing

Transcriptome RNA sequencing (RNA-seq) data of ccRCC samples were obtained from The Cancer Genome Atlas (TCGA). The RNA-seq data selected for analysis were normalized using the Fragments Per Kilobase of Transcript Per Million Mapped Reads (FPKM) method. After eliminating duplicated data entries and samples with missing information, the final TCGA-KIRC cohort comprised 29 normal samples and 400 cancer samples, of which 141 were from females and 259 were from males, aged 68.0 ± 10.64 years. All patients had accompanying clinical information.

The T1DM peripheral blood mononuclear cell (PBMC) transcriptome dataset GSE55098, the T1DM validation cohort GSE9006, and the ccRCC transcriptome validation cohort GSE53757 were obtained from the GEO database. The GPL96 and GPL97 platforms from the UTSW Medical Center were used for GSE9006. Our study used data based on the GPL97 platform, including 24 normal PBMC samples and 43 PBMC samples from T1DM patients (26 women and 17 men; aged 10.1 ± 3.8 years). The GPL97 platform from Shanghai JiaoTong University Ruijin Hospital was used for GSE53757, which included 10 normal PBMC samples and 12 PBMC samples from patients with T1DM (5 women and 7 men; aged 17.5 ± 3.68 years). The GPL570 platform from The Mayo Clinic was used for GSE53757, which consists of 72 normal samples and 72 ccRCC samples. Details of the dataset are shown in **Supplementary Table S1**. The demographic characteristics of the TCGA-KIRC cohort involved in this study are shown in **Supplementary Table S4**

### 2.2 Weighted gene coexpression network analysis

We chose WGCNA as the analysis method for the T1DM transcriptome data because this method can identify coexpressed gene modules closely related to T1DM as a whole, revealing the pathway by which T1DM influences the development and prognosis of ccRCC. We employed the “WGCNA” package (17) to construct a weighted adjacency matrix by selecting appropriate thresholds. Subsequently, the weighted adjacency matrix was transformed into a topological overlap matrix (TOM). To identify modules with the most significant correlation with T1DM, we applied the dynamic tree-cutting algorithm to divide the network into distinct modules and extracted the genes within the modules.

### 2.3 Identification of differentially expressed genes and ccRCC prognostic genes

The “limma” package (18) was used to identify differentially expressed genes (DEGs) between normal and diabetic samples in the GSE55098 dataset and between normal and tumor samples in the TCGA-KIRC cohort. Genes with an adjusted p value < 0.05 and |log2FC|>1 were considered DEGs. Then, we performed univariate Cox regression analysis using the “survival” package to identify prognostic genes (p value < 0.05). Genes meeting the criteria of both DEGs in ccRCC, specific prognostic genes for ccRCC, and present in the modules most strongly correlated with T1DM were classified as TIDM-related prognostic DEGs for subsequent analysis.

### 2.4 GeneMANIA-associated analysis and PPI network construction

Due to the limited number of TIDM-related prognostic DEGs, directly applying them to machine learning models may lead to unreliable results. Drawing on the approach adopted in previous studies (19–21), the GeneMANIA database (22) was used to identify genes associated with TIDM-related prognostic DEGs. The STRING database (23) and Cytoscape were used to construct a protein‒protein interaction (PPI) network.

### 2.5 Functional enrichment analysis

To investigate the pathways shared between T1DM and ccRCC, we performed Gene Ontology (GO) term enrichment and Kyoto Encyclopedia of Genes and Genomes (KEGG) pathway analyses using the Metascape database. These analyses were based on T1DM-related prognostic DEGs and their associated genes (24).

### 2.6 Hub gene screening and validation based on the machine learning algorithm

We applied three machine learning methods to identify key biomarkers associated with ccRCC prognosis from T1DM-associated ccRCC prognostic DEGs and their associated genes based on the TCGA-KIRC cohort. First, we evaluated the efficiency of the random forest (RF) and support vector machine recursive feature elimination (SVM-RFE) algorithms in the TCGA-KIRC cohort through residual and receiver operating characteristic (ROC) curve analyses. We then selected the genes identified by one of these algorithms. It is important to clarify that our study’s machine learning models were trained using the presence or absence of renal cancer as the response variable, with 20 ccRCC biomarkers related to T1DM as covariates. Notably, we utilized the “randomForest” package (25) for constructing the random forest model and the “Kernlab” package (26) for constructing the SVM-RFE model. The random forest model employed default feature selection based on mean decrease impurity, while the SVM-RFE model employed recursive feature impurity removal for feature selection. We implemented K-fold cross-validation for feature extraction, ensuring that both the SVM/RF and least absolute shrinkage and selection operator (LASSO) models utilized this data splitting method for accurate and reliable feature comparisons. Subsequently, LASSO regression was performed, and we selected one of the algorithms according to the advantages and disadvantages of RF or SVM-RFE and intersection with the genes found in LASSO regression analysis as the hub genes.

### 2.7 Evaluation of the prognostic efficacy of the hub genes

The prognostic efficacy of the hub genes in T1DM patients and ccRCC patients was evaluated by constructing ROCs and calculating the corresponding area under the curve (AUC) using the “pROC” package. After grouping based on the cutoff values calculated by the “survival” package, Kaplan‒Meier survival curves corroborated a substantial correlation between the hub genes and the survival outcomes of ccRCC patients.

### 2.8 Gene set enrichment analysis of the hub genes in patients with T1DM and ccRCC

GSEA is a computational method used to determine whether predefined sets of genes show statistically significant, coordinated differences between two biological states. We conducted single-gene GSEA of the hub genes in the T1DM cohort GSE55098 and TCGA-KIRC cohort using the “fgsea” package (27), with the hallmark gene set obtained from the MsigDB database (28, 29). Specifically, samples were divided into high- and low-expression groups based on the expression levels of the hub genes. Differential expression analysis was then conducted to identify DEGs between these two groups. GSEA was subsequently applied to evaluate the association between the target genes and specific biological processes or pathways.

### 2.9 Assessing the role of the hub genes in the tumor microenvironment

The “Cibersort” package (30) was used to analyze the abundance ratios of 22 types of immune cells in the TCGA ccRCC cohort and to evaluate the interactions between different immune cells. Furthermore, the associations between the hub genes and specific immune cells were investigated.

### 2.10 Single-cell RNA sequencing analysis

We obtained ccRCC scRNA-seq data comprising seven tumor samples and two adjacent normal tissue samples from the GSE210042 dataset in the GEO database. In this study, we only used single-cell data from tumor samples. Data preprocessing was conducted using the “Seurat” package (version 4.1.0) (31) with the following criteria for the tumor samples: nFeature_RNA > 200, nCount_RNA > 1000, and fewer than 20% of mitochondrial genes. A total of 50,487 cells were obtained for subsequent analysis. The top 2000 highly variable genes were identified using the “FindVariableGenes” function, and principal component analysis was subsequently conducted. Cell types were annotated based on known renal-specific marker genes from a previous study (32) or queried in PanglaoDB (33) to assign cell types based on cluster-specific marker genes. Pseudotime analysis was performed using the “monocle3” package (34). We examined hub gene expression across all cell clusters and explored cellular crosstalk using the “CellChat” package (35).

### 2.11 Human renal RNA extraction and quantitative real-time polymerase chain reaction

Tumor and adjacent normal tissues were collected from 12 ccRCC patients who underwent radical nephrectomy, and RNA was extracted from these tissues. This study was approved by the Ethics Committee of the First Affiliated Hospital of Jinan University, and written informed consent was obtained from all patients and control individuals.

Total RNA was extracted using the EZ-Press RNA Purification Kit (EZbioscience, USA). cDNA was synthesized using the PrimeScript RT Kit (TaKaRa, Japan) through reverse transcription. RT–qPCR detection was performed using a CFX96 real-time PCR system (Bio-Rad, USA) following the SYBR Green method (Vazyme, China). After normalization to GAPDH expression, relative mRNA expression levels were determined. The mRNA-specific primer sequences can be found in **Supplementary Table S2**.

### 2.12 Statistical analysis

Statistical analyses were conducted using R 4.1.1 and GraphPad Prism 8 (GraphPad Software, Inc.), results are based on two-group comparisons, and no multiple-group comparisons were performed. Continuous variables were described as the mean standard deviation (SD) when normally distributed or median (interquartile range, IQR) when not, and categorical variables were described as numbers (percentage). The means of medians for continuous variables in two independent groups were tested by the Wilcoxon rank-sum test. The enumeration data analyzed by using t-test. p<0.05 was considered statistically significant. The error bars indicate the standard deviation in the figures.

## 3 Results

### 3.1 Identification of T1DM-related prognostic DEGs

**Figure 2:**
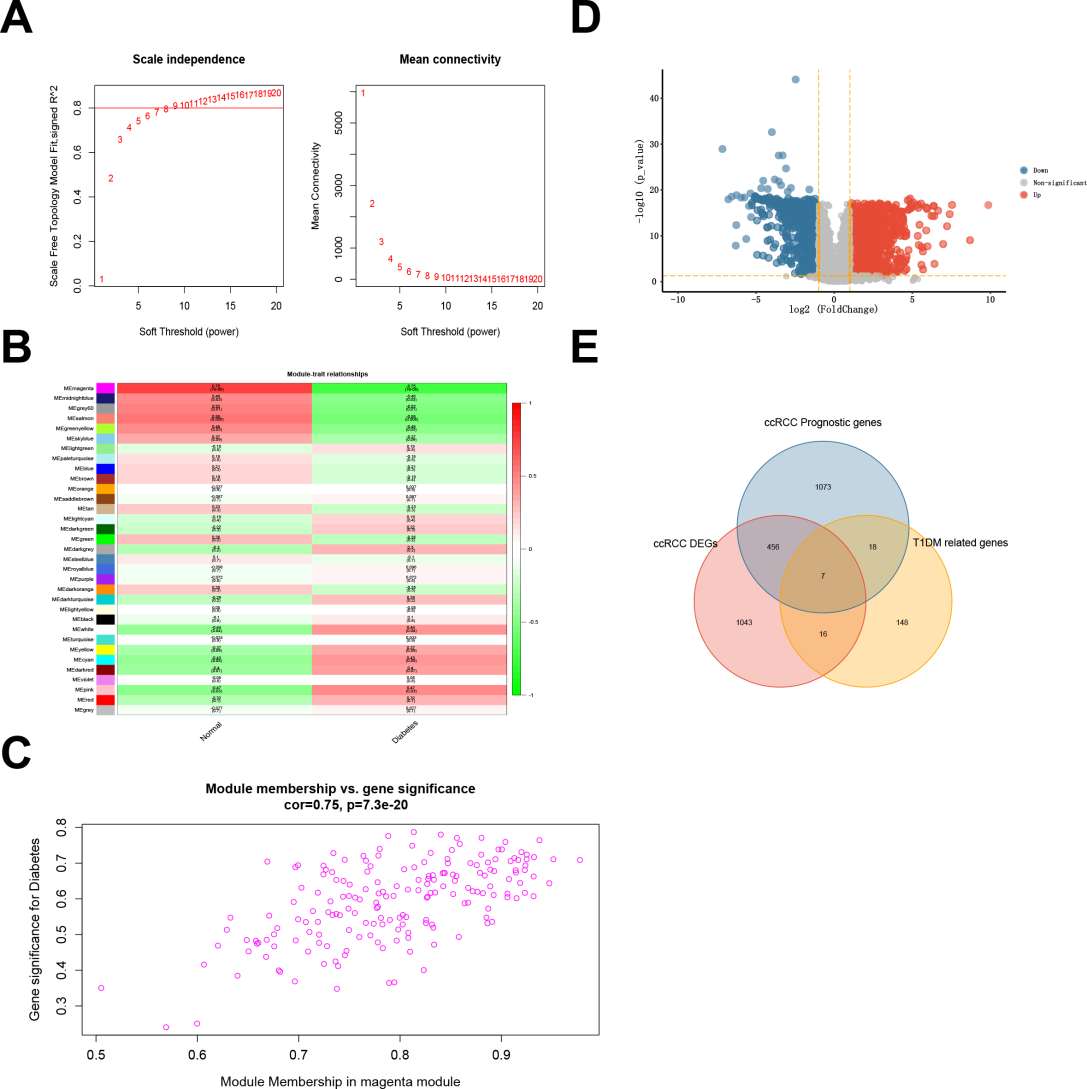
**(A)** Fitting index of the scale-free topology module under different soft thresholds (left) and network connectivity under different soft thresholds (right). **(B)** Correlations between module eigengenes and type 1 diabetes. **(C)** Scatterplot showing the relationship between gene significance (GS) for type 1 diabetes and module membership (MM) in the magenta module. **(D)** Differentially expressed genes (DEGs) in ccRCC samples compared with normal samples in the TCGA-KIRC cohort. **(E)** Intersection between the DEGs and prognostic genes from the TCGA-KIRC cohort and the magenta module genes from the T1DM GEO dataset.

WGCNA was used to determine an optimal soft threshold (β = 9) for constructing a scale-free network (**Figure 2A**) after multiple iterations. After excluding the gray module, which contained genes not involved in clustering, we identified 32 modules. Correlation analysis between these modules and T1DM highlighted the magenta module, consisting of 189 genes, as having the highest correlation with T1DM (correlation coefficient = 0.75, P < 0.001) (**Figure 2B**). A scatterplot further illustrates the correlation coefficient (0.75, P < 0.001) between GS and MM within the magenta module (**Figure 2C**).

In the TCGA-KIRC cohort, we identified 1521 DEGs, comprising 947 upregulated and 574 downregulated DEGs (**Figure 2D**; **Supplementary Table S3**). Subsequently, 1554 prognosis-related genes were identified through univariate Cox regression. The intersection of DEGs, prognostic genes, and magenta module genes revealed seven genes designated as TIDM-related prognostic DEGs (**Figure 2E**).

### 3.2 Identification of genes associated with the prognosis of patients with TIDM and enrichment analysis

**Figure 3:**
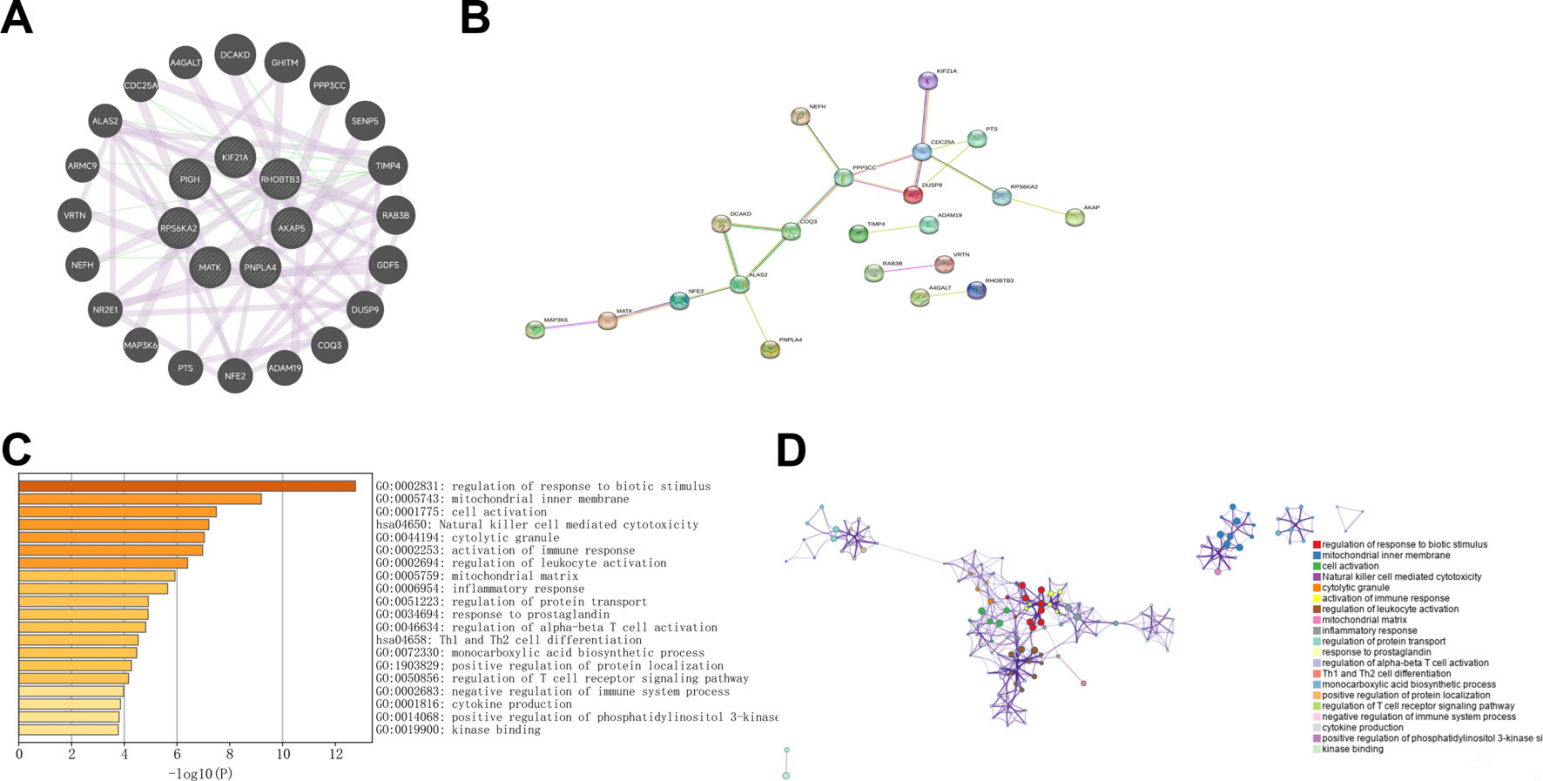
**(A)** GeneMANIA was utilized to identify the interacting genes associated with T1DM-related prognostic differentially expressed genes (DEGs) in clear cell renal carcinoma (ccRCC). **(B)** The protein‒protein interaction (PPI) network visualizes the interactions between T1DM-related prognostic DEGs and their interacting genes in ccRCC. **(C, D)** KEGG and GO enrichment analyses of T1DM-related prognostic DEGs and their interacting genes in ccRCC.

A set of 20 genes associated with the TIDM-related prognostic DEGs was identified using GeneMANIA (**Figure 3A**). Subsequently, a PPI network was visualized through Cytoscape (**Figure 3B**). To gain insight into the functional implications of these genes, enrichment analysis was performed via the Metascape database. The results indicated that the following pathways may represent pathways shared between T1DM patients and ccRCC patients: the mitogen-activated protein kinase (MAPK) signaling pathway, the biosynthesis of cofactors, protein domain-specific binding, phosphotransferase activity, and the modulation of chemical synaptic transmission (**Figures 3C, D**).

### 3.3 Selection of shared biomarkers between T1DM patients and ccRCC patients using multiple machine learning methods

We first used the RF and SVM-RFE models for gene selection. **Supplementary Figure S1A** shows gene selection based on the minimum error point in the RF model (X=152). The RF method exhibited superior machine learning capabilities, as evidenced by smaller residuals (**Supplementary Figures S1B–C**) and higher AUC values (**Supplementary Figure S2A**) than those of the SVM. Consequently, leveraging the RF method, we identified seven candidates from 27 T1DM-related prognostic differentially expressed genes (DEGs) and their associated genes. Furthermore, LASSO regression showed high AUC values in the TCGA-KIRC cohort (**Supplementary Figure S2B**), and identified nine genes with the lowest binomial deviance (**Supplementary Figures S1D, E**). The intersection of genes detected by both the RF method and LASSO regression revealed three hub genes, KIF21A, PIGH, and RPS6KA2, as illustrated in a Venn diagram (**Supplementary Figure S1F**).

### 3.4 Validation of the expression patterns of the hub genes

**Figure 4:**
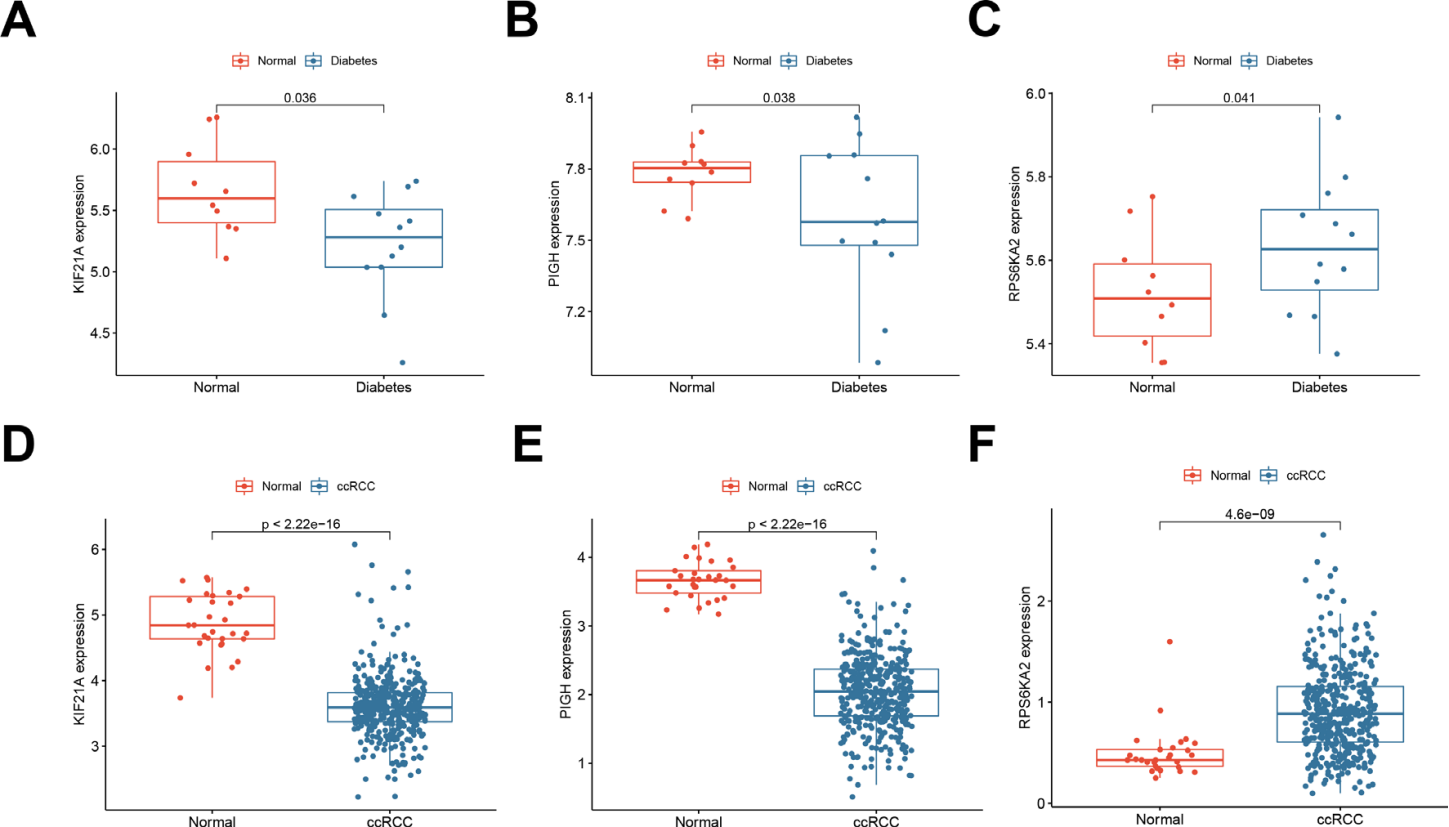
**(A-C)** Expression of the hub genes KIF21A, PIGH, and RPS6KA2 in normal samples and T1DM samples (GSE55098). **(D-F)** Expression of the hub genes KIF21A, PIGH, and RPS6KA2 in normal samples and ccRCC samples (TCGA-KIRC).

We validated the expression of three hub genes in both T1DM patients and ccRCC patients. Similarly to PIGH, KIF21A was expressed at low levels in both T1DM (**Figure 4A**) and ccRCC (**Figure 4D**) patients (**Figures 4B, E**). Conversely, the opposite trend was observed for RPS6KA2 (**Figures 4C, F**).

**Figure 5:**
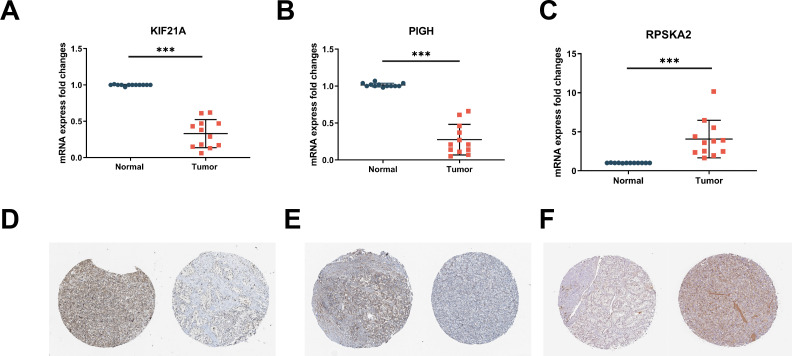
**(A-C)** The mRNA expression levels of KIF21A **(A)**, PIGH **(B)**, and RPS6KA2 **(C)** in clinical samples were detected by RT‒qPCR. **(D-F)** Immunohistochemistry of KIF21A **(D)**, PIGH **(E)**, and RPS6KA2 **(F)** in normal tissues (left) and ccRCC tissues (right) from the HPA database. *** indicates P < 0.001.

To address the issue of patient diversity, the study analyzed the demographic characteristics of the TCGA-KIRC cohort (**Supplementary Table S4**), which primarily includes White and Black patients. While no significant effects of these factors on gene expression were observed, however, the potential influence of ethnicity on disease mechanisms should not be ignored. Thus, to mitigate this limitation, we validated our findings using RT-qPCR on clinical samples from Asian patients, demonstrating the applicability of our results across diverse populations. This partially addresses the underrepresentation in public datasets. By RT‒qPCR, we confirmed the downregulation of KIF21A (**Figure 5A**) and PIGH (**Figure 5B**) and the upregulation of RPS6KA2 (**Figure 5C**) in ccRCC tissues compared to normal tissues. Immunohistochemical (IHC) analysis of samples from the Human Protein Atlas (HPA) database further confirmed these trends: KIF21A (**Figure 5D**) and PIGH (**Figure 5E**) exhibited low expression, while RPS6KA2 (**Figure 5F**) exhibited high expression in ccRCC tissues compared to normal tissues. These findings provide strong evidence for consistent expression patterns of these three hub genes in both T1DM patients and ccRCC patients.

### 3.5 Validation of the prognostic efficacy of the hub genes

**Figure 6:**
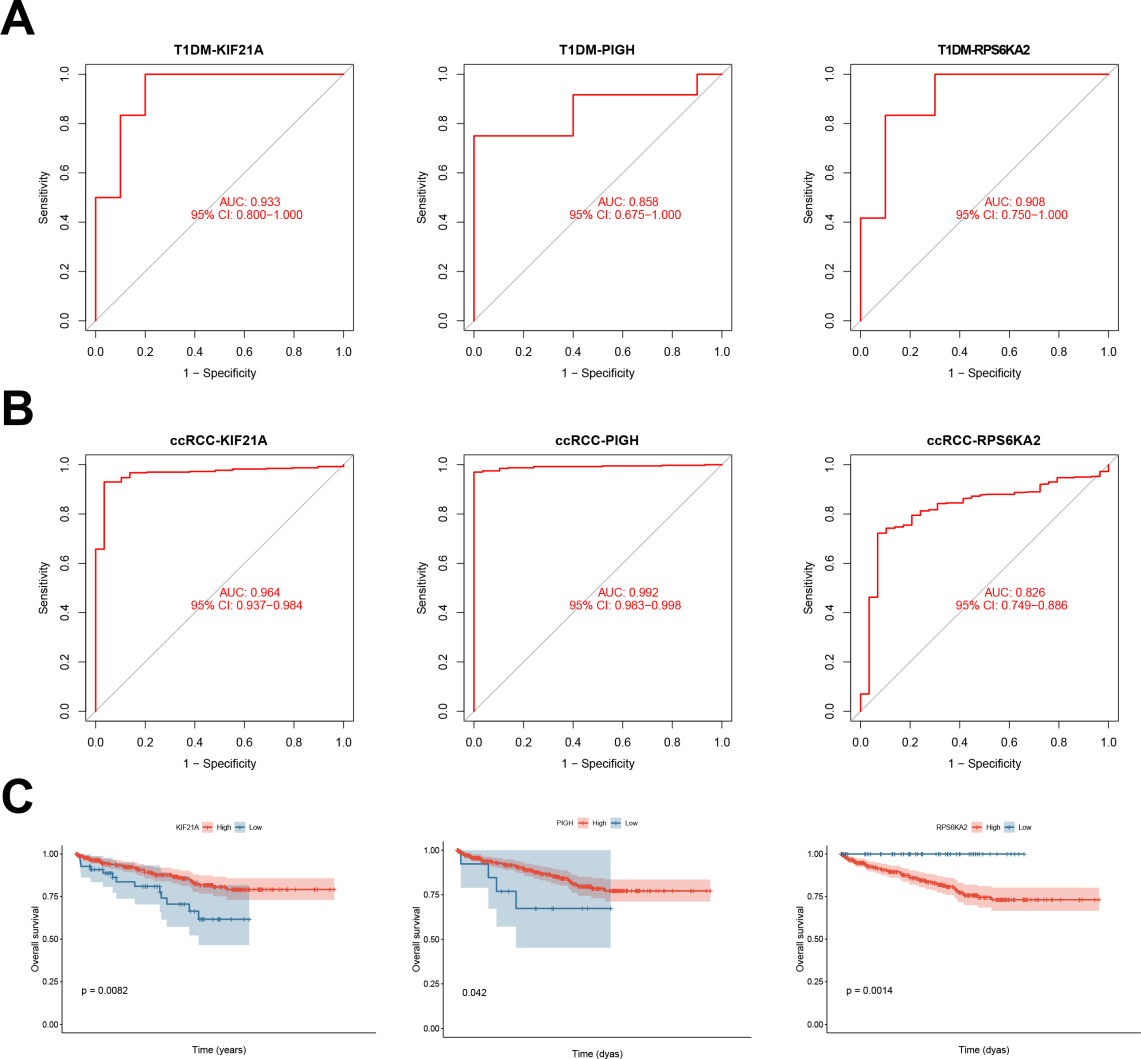
**(A)** ROC curves showing the prognostic performance of KIF21A, PIGH, and RPS6KA2 in patients with T1DM. **(B)** ROC curves depicting the prognostic performance of KIF21A, PIGH, and RPS6KA2 in ccRCC. **(C)** Kaplan‒Meier survival curves illustrating the prognostic impact of KIF21A, PIGH, and RPS6KA2 in ccRCC patients.

Univariate Cox regression analysis was performed to validate the prognostic significance of the hub genes in both T1DM patients and ccRCC patients. KIF21A, PIGH, and RPS6KA2 demonstrated significant prognostic performance in T1DM patients, with AUC values of 0.933, 0.858, and 0.908, respectively (**Figure 6A**). In ccRCC patients, the AUC values were 0.964, 0.992, and 0.826, respectively (**Figure 6B**). K‒M survival curves confirmed the significant correlation between these genes and the survival of ccRCC patients (**Figure 6C**).

**Figure 7:**
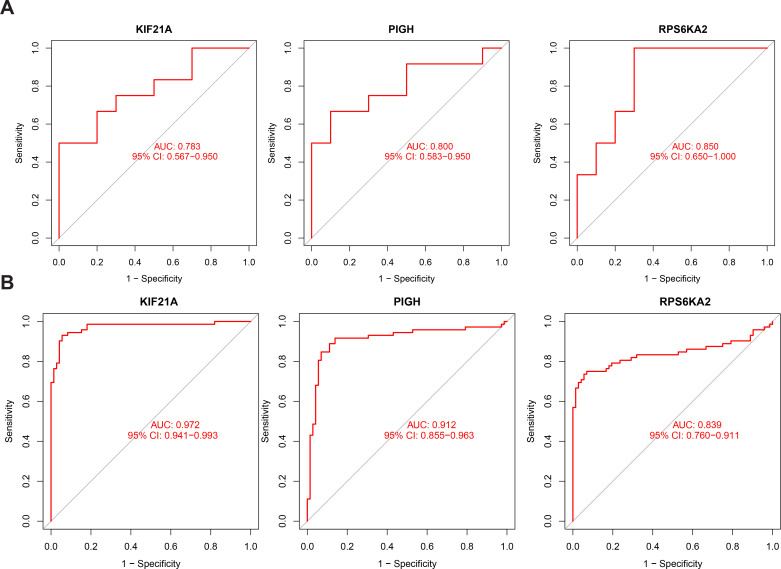
**(A)** ROC curve of KIF21A, PIGH, and RPS6KA2 in the validation cohort for T1DM. **(B)** ROC curve of KIF21A, PIGH, and RPS6KA2 in the validation cohort for ccRCC.

We also assessed the prognostic performance of the hub genes in independent validation datasets for both T1DM (GSE9006) and ccRCC (GSE53757) patients. In T1DM patients, the AUC values for KIF21A, PIGH, and RPS6KA2 were 0.783, 0.800, and 0.850, respectively (**Figure 7A**). In ccRCC patients, the AUC values were 0.972, 0.912, and 0.839, respectively (**Figure 7B**). Combining the results from the original and validation cohorts, it is evident that these hub genes exhibit superior prognostic efficacy in both T1DM patients and ccRCC patients.

### 3.6 Single-gene GSEA of the hub genes in patients with T1DM and ccRCC

We performed single-gene GSEA of the hub genes in the T1DM and ccRCC cohorts.

**Figure 8:**
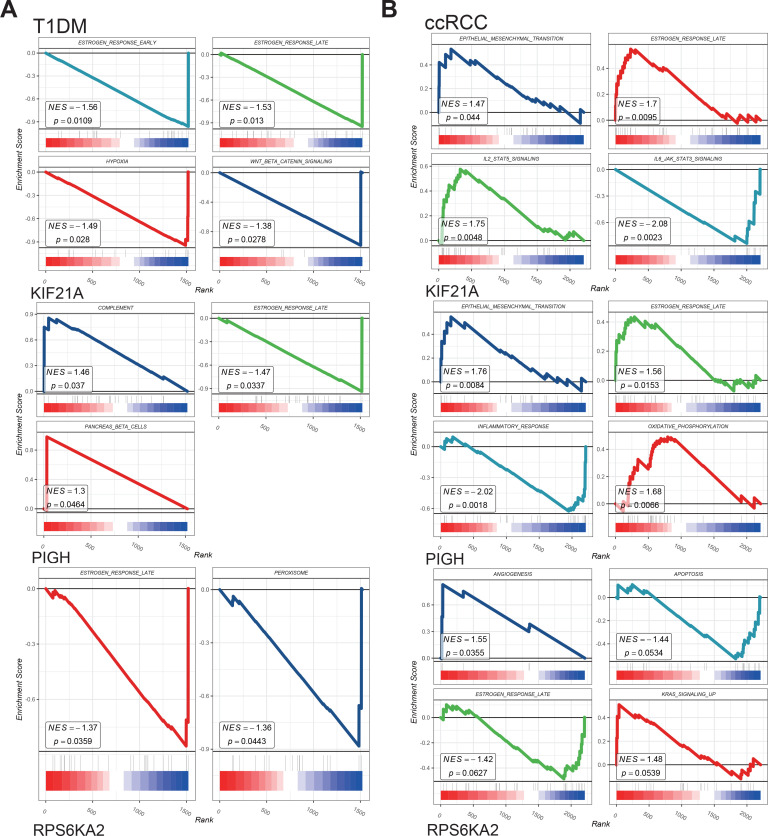
**(A)** GSEA analysis for hub genes in T1DM. **(B)** GSEA analysis for hub genes in ccRCC.

The results showed that the hub genes were involved in the estrogen response, epithelial–mesenchymal transition, inflammatory response, angiogenesis, KRAS signaling, and other pathways in both disease groups (**Figures 8A, B**). The identified pathways highlight shared mechanisms underlying T1DM and ccRCC, supporting KIF21A, PIGH, and RPS6KA2 as shared biomarkers. Such as estrogen signaling enhances insulin-like growth factor-1 receptor activity, exacerbating the effects of elevated insulin and promoting ccRCC development (36). Increased oxidative phosphorylation activates the pyruvate dehydrogenase complex, reprogramming glucose metabolism and the TCA cycle, thereby facilitating tumor growth and metastasis (37). Additionally, partial complement system activation increases immune cell infiltration in the tumor microenvironment while promoting immune evasion, ultimately driving distant metastasis (38). These shared pathways underscore the interconnectedness of T1DM and ccRCC, with dysregulated mechanisms contributing to ccRCC risk and progression in T1DM patients. The roles of KIF21A, PIGH, and RPS6KA2 in these pathways reinforce their potential as diagnostic and therapeutic biomarkers.

### 3.7 Associations between immune cell infiltration and hub genes in ccRCC

**Figure 9:**
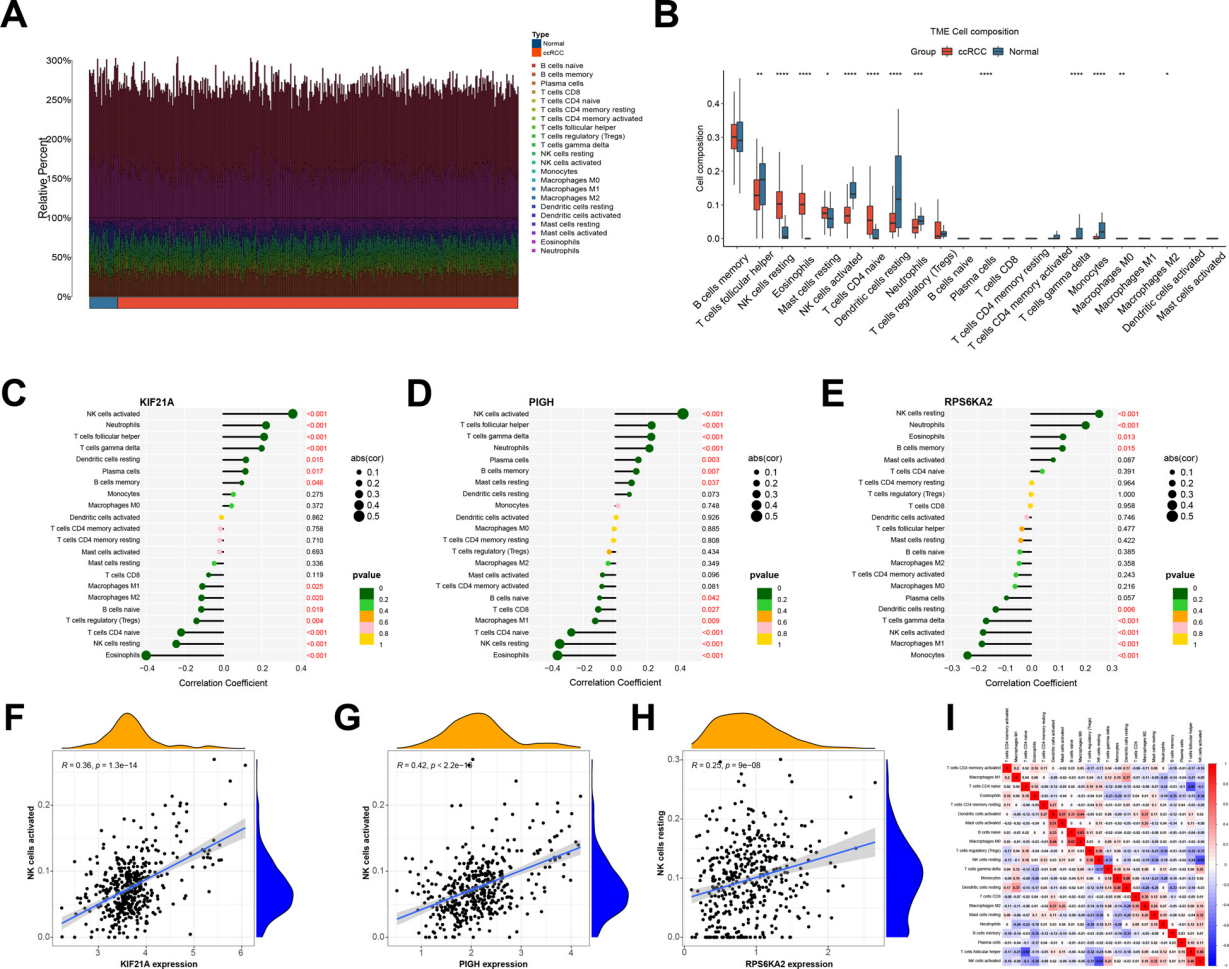
**(A)** Immune cell infiltration of the TCGA-KIRC cohort. **(B)** Differences in immune cell abundance between the normal and ccRCC groups. **(C-E)** Correlations between hub genes and various immune cell populations. **(F-H)** A significant correlation was observed between the expression of the hub genes and NK cell infiltration. **(I)** The correlation between immune cells. *indicates P < 0.05, ** indicates P < 0.01, *** indicates P < 0.001, **** indicates P < 0.0001.

We compared immune cell infiltration between the normal and ccRCC groups in the TCGA-KIRC cohort (**Figure 9A**). Most immune cells, including memory B cells, NK cells, T helper cells, dendritic cells, and monocytes, exhibited increased infiltration in the normal group. Conversely, immunosuppressive cells such as Tregs and resting immune cells were more abundant in the ccRCC group (**Figure 9B**).

We also examined the correlation between the hub genes and immune cells infiltrating ccRCC. KIF21A and PIGH exhibited positive correlations with activated NK cells, follicular helper T cells, and γδ T cells, while they had negative correlations with eosinophils, resting NK cells, naive CD4 T cells, and Tregs, and RPS6KA2 was positively correlated with NK cells resting. It was negatively correlated with NK cells activated and M1 macrophages (**Figures 9C–E**). Notably, there was a significant positive association between KIF21A and PIGH with activated NK cells, which are immune cells with broad-spectrum anti-tumor effects, while RPS6KA2 was negatively correlated with its activation (**Figures 9F–H**). Given the low expression of KIF21A and PIGH in ccRCC and the high expression of RPS6KA2, KIF21A and PIGH may act as tumor suppressor genes that promote NK cell activation in ccRCC, while RPS6KA2 may function as an oncogene that inhibits NK cell activation.

Finally, we analyzed the interactions between different immune cells and revealed positive correlations between activated NK cells and TFH cells, γδ T cells, and resting mast cells but negative correlations between eosinophils and naive CD4 T cells (**Figure 9I**).

### 3.8 Single-cell sequencing analysis

**Figure 10:**
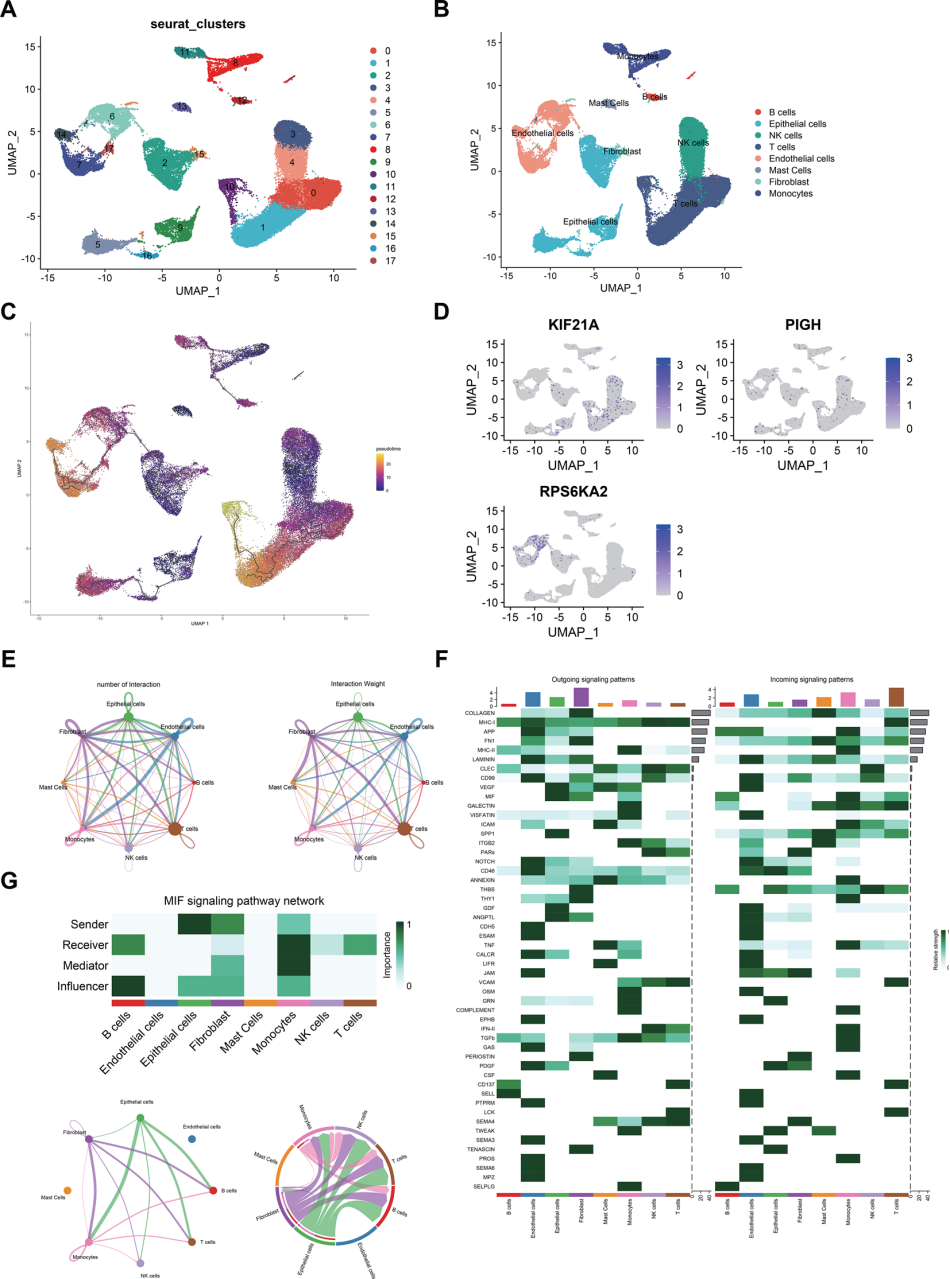
**(A)** UMAP visualization of 50,487 cells in the single-cell RNA sequencing dataset GSE21002, where distinct colors indicate different cell clusters. **(B)** Eight cell clusters (B cells, epithelial cells, NK cells, T cells, endothelial cells, mast cells, fibroblasts, and monocytes) were identified on the UMAP plot. **(C)** Potential trajectory of eight cell clusters. **(D)** Expression levels of hub genes across different cell clusters. **(E)** The number and strength of interactions between cell populations. **(F)** Various intercellular signaling pathways in which different populations participate. **(G)** The MIF signaling networks.

After quality control, we analyzed the ccRCC microenvironment using scRNA-seq data, which involved clustering 40215 cells into 18 distinct clusters (**Figure 10A**). These 18 clusters were further categorized into eight cell populations through cell cluster annotation (**Supplementary Figure S1**) (**Figure 10B**). Pseudotime analysis revealed the pseudotime trajectory of these cell populations (**Figure 10C**). Our single-cell analysis showed that KIF21A was primarily localized in NK and T cells, PIGH was mainly expressed in NK, T, and endothelial cells, and RPS6KA2 targeted epithelial and endothelial cells (**Figure 10D**).

We also investigated interactions among these eight cell populations and noted a strong correlation between NK cells and CD8+ T cells (**Figure 10E**). CellChat analysis revealed interaction patterns among tumor, immune, and stromal cells in the ccRCC microenvironment. In pathways such as the SPP1, THBS, and GDF pathways, epithelial cells act as prominent senders, while NK cells and T cells act as receivers. Conversely, in the CD99 pathway, NK and T cells were senters, and epithelial cells were receivers (**Figure 10F**). Notably, in the macrophage migration inhibitory factor (MIF) pathway, epithelial cells were the strongest sender, indicating robust MIF pathway activity between epithelial cells, NK cells, and T cells (**Figure 10G**).

## 4 Discussion

Currently, the precise pathological mechanism underlying ccRCC remains elusive; however, gaining insights into the mechanisms driving the initiation of ccRCC is essential for formulating effective prevention strategies. Despite its relatively low incidence in the population, however, the onset of ccRCC is insidious, and its propensity for metastasis intensifies in advanced stages. Even after surgical resection, the recurrence rate of ccRCC is approximately 20-40%. For those with metastatic ccRCC, despite the combination of chemotherapy, the prognosis remains grim (39). Therefore, early detection and prevention of ccRCC has important clinical significance, especially for high-risk groups such as diabetic patients.

T1DM is an autoimmune disorder characterized by the progressive destruction of β cells (5). The human leukocyte antigen risk alleles present islet antigens to CD4+ T cells, initiating an immune response against β cells. Additionally, CD8+ T cells are stimulated by cytokines produced by B and T cells (40, 41). Consequently, as the primary cytotoxic effectors in specific immune responses, CD8+ T cells are pivotal for destroying β cells during the progression from insulitis to T1DM (42).

Recent studies indicate that the incidence of T1DM is progressively increasing; while it predominantly develops in children and young adults, many patients are diagnosed with this condition during adulthood. In such cases, the destruction of β cells is variable, exhibiting characteristics of both T1DM and T2DM. This unique manifestation may be associated with the penetrance (43, 44) of the immune system. Notably, long-term DM is correlated with an elevated risk of numerous cancers (45). In patients with long-term DM, metabolic syndrome mainly includes obesity and high blood sugar. Obesity triggers changes in the immune microenvironment, including hyperinsulinemia, elevated insulin-like growth factor levels, and chronic inflammation, which collectively contribute to cancer progression (46). Many cancers, including ccRCC, rely on increased carbohydrate metabolism. Cancer cells in hyperglycemic patients can absorb more glucose, leading to increased proliferation, metastasis, and poor prognosis (47), and hyperglycemia induces oxidative stress and DNA damage, promoting genomic instability and cancer initiation (48). Thus, as a chronic proinflammatory condition, DM significantly affects the tumor microenvironment, promoting cancer occurrence (49).

By integrating multiple transcriptome data and ccRCC scRNA-seq data, we used multiple machine learning methods to screen three biomarkers that are common between T1DM patients and ccRCC patients (KIF21A, PIGH, and RPS6KA2), which are linked to tumor and immune infiltration, and to predict the trajectory and prognosis of T1DM patients and ccRCC patients in an independent validation cluster. Our results revealed multiple pathways linking T1DM with ccRCC, which may be related to increased inflammation and an adverse prognosis for patients with ccRCC. For example, in the MAPK pathway, both diseases may involve several cascade protein kinases that coordinate intracellular signaling. This pathway influences lymphocyte differentiation, activation, and inhibition (50). Overactivation of the MAPK pathway could promote the transformation of renal cysts to ccRCC and enhance the proliferation of cancer cells, thereby enhancing the progression of the disease (51).

Among these biomarkers, KIF21A plays a pivotal role in microtubule assembly, and research has shown that its activity is significantly decreased in several cancers, subsequently influencing the regulation of cell polarity and migration (52). Previous investigations have shed light on the role of KIF21A in lung cancer. The expression of KIF21A decreases owing to DNA methylation in patients with lung cancer. This decrease in expression heightens the risk of distant metastasis of the cancer (53).

PIGH is an anchoring mechanism for numerous cell membrane-bound proteins and plays an integral role in synthesizing glycosyl phosphatidylinositol (54). Downregulation of PIGH expression leads to heightened chemotherapy resistance and bolsters the phenomenon of immune escape within the realm of cancer (55).

RPS6KA2, a serine/threonine protein kinase family member, is substantially overexpressed in several cancer types, including prostate, breast, and pancreatic cancers. Intriguingly, such elevated RPS6KA2 levels have been associated with fortified drug resistance and the reversal of cancer cell apoptosis typically induced by chemotherapy (56).

Consistent with the above conclusion, our study showed that ccRCC patients with low KIF21A and PIGH expression and elevated RPS6KA2 levels exhibit a poorer prognosis and overall survival than their low-risk counterparts. The analysis of the scRNA-seq data revealed that KIF21A and PIGH predominantly localize to NK cells, CD4+ T cells, and CD8+ T cells, and decreased expression of these genes may reduce the activation of NK cells and reduce the number of NK cells in patients with T1DM, increasing the risk of cancer onset (6, 57). RPS6KA2 is mainly localized in endothelial and epithelial cells. Single-gene GSEA revealed that RPS6KA2 was significantly enriched in pathways associated with angiogenesis and KRAS signaling in the TCGA cohort with high RPS6KA2 expression. Therefore, RPS6KA2 may play a role in two ways: on the one hand, it may promote endothelial cell angiogenesis, and on the other hand, it may promote the progression of malignant epithelial cells by activating the KRAS signaling pathway; on the other hand, in diabetic patients, hyperglycemia and inflammation trigger endothelial dysfunction, which may eventually lead to vascular remodeling and renal damage (58). Given that RPS6KA2 is localized primarily within endothelial cells, RPS6KA2 may play a role in transforming tumor-derived endothelial cells into cancer cells and promoting vascularization. The vascularization process is closely related to tumor proliferation and metastasis (59). Thus, RPS6KA2 may be essential for multiple vital steps in tumor development. Moreover, we used CellChat to investigate cell interactions within the ccRCC microenvironment. Our findings revealed that epithelial cells, NK cells, and T cells all play active roles in the MIF signaling pathway, indicating their potential involvement in mediating interactions among these cell types.

This study focuses on exploring potential associations between T1DM-related genes and ccRCC risk, laying the groundwork for future mechanistic research. While it remains unclear whether genes such as KIF21A, PIGH, and RPS6KA2 directly drive disease processes or are secondary to other factors, we propose that their dysregulation may result from the proinflammatory and metabolic changes associated with T1DM. This dysregulation likely impacts shared biological pathways, including estrogen response, oxidative phosphorylation, and signal transduction, thereby increasing ccRCC risk or accelerating its progression. Moreover, the proinflammatory state in T1DM may exacerbate gene dysregulation, further driving processes that promote ccRCC development and progression.

We validated the prognostic value of these markers in both the original and independent datasets, confirming their importance in ccRCC patient survival and management. Although no significant effect of race on hub gene expression was observed, the potential impact of race on disease mechanisms should not be overlooked. Thus, to mitigate this limitation, we validated our findings using RT-qPCR on clinical samples from Asian patients, demonstrating the applicability of our results across diverse populations. This partially addresses the underrepresentation in public datasets.

The strong association of these hubgenes with ccRCC prognosis and the stability of their expression across ethnic groups highlight their potential as biomarkers for early detection and personalized patient management. We propose incorporating these biomarkers into liquid biopsy platforms, such as ctDNA or RNA-based assays, using advanced techniques like digital PCR and NGS to enable early, non-invasive detection of ccRCC, especially in high-risk populations like T1DM patients.

Due to our study’s reliance on bioinformatics methods, issues with sample quality and batch effects within public databases may have introduced bias in the analysis results. Consequently, additional *in vivo* or *in vitro* experiments are essential to further validate our findings. In this study, distinct external validation sets were utilized for ccRCC and T1DM patients. However, it is imperative to substantiate the robustness of the hub genes by incorporating more external datasets in future investigations. Furthermore, our conclusions are drawn from diverse patient cohorts and lack validation within the same individual. Thus, future research should employ animal models that integrate ccRCC and T1DM to elucidate the potential relationship between these two diseases.

## 5 Conclusion

In this study, T1DM-related genes were identified using WGCNA, and three hub genes (KIF21A, PIGH, and RPS6KA2) were selected through feature selection in machine learning models that intersected with ccRCC prognostic DEGs; we also explored their role and potential mechanism in the development of ccRCC. Single-cell RNA sequencing analysis has laid the groundwork for obtaining a comprehensive understanding of the functions and interactions of these biomarkers within the immune microenvironment. This work is anticipated to offer possibilities for the prevention or early detection of ccRCC in T1DM patients and to provide novel targets for new pharmacotherapies.

## Data Availability

The datasets presented in this study can be found in online repositories. The names of the repositories and accession numbers are provided in **Table S1**. Specifically, the T1DM peripheral blood mononuclear cell transcriptome dataset (GSE55098), the T1DM validation cohort (GSE9006), and the ccRCC transcriptome validation cohort (GSE53757) were obtained from the Gene Expression Omnibus (GEO) database (https://www.ncbi.nlm.nih.gov/geo/). Additionally, the TCGA-KIRC cohort was obtained from The Cancer Genome Atlas (TCGA) (https://portal.gdc.cancer.gov/). All datasets used in this study are publicly available in these databases, they can be accessed at any time.

## Supplementary material

Supplementary Figure 1
**(A)** Gene selection using the random forest algorithm. **(B)** Comparison of the reverse cumulative residual distribution between the random forest and support vector machine methods. **(C)** Comparison of the residual values between the random forest and SVM-RFE methods; the red dots represent the root mean square of the residuals. **(D)** Selection of the optimal parameter (λ) for LASSO regression through cross-validation. **(E)** Gene selection using LASSO regression. **(F)** The hub genes were selected as the intersection of genes identified by the random forest and LASSO algorithms. * RF, SVM-RFE, and LASSO analyses were performed using the TCGA-KIRC cohort.

Supplementary Figure 2
**(A)** Comparison of the receiver operating characteristic (ROC) curves between the random forest and SVM methods in TCGA-KIRC cohort. **(B)** The ROC curve of LASSO method in TCGA-KIRC cohort.

Supplementary Figure 3 Bubble plot depicting marker gene expression levels in each cell type.
