# Supplementary figures and images for: Integrating machine learning and single-cell sequencing to identify shared biomarkers in type 1 diabetes mellitus and clear cell renal cell carcinoma
Source: Front Oncol. 2025 Mar 3;15:1543806. doi: 10.3389/fonc.2025.1543806 (PMC11911197; doi:10.3389/fonc.2025.1543806)

**A**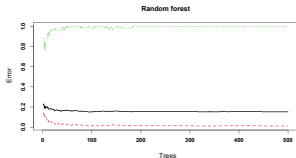**B**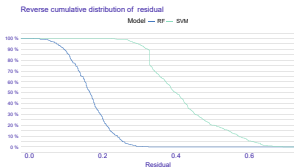**C**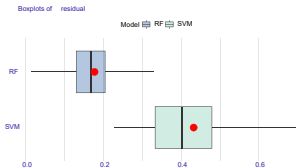**D**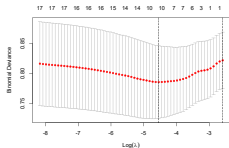**E**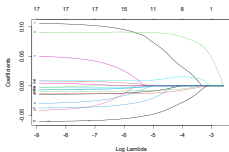**F**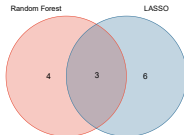

Supplement: Supplementary Figure 1 — (A) Gene selection using the random forest algorithm. (B) Comparison of the reverse cumulative residual distribution between the random forest and support vector machine methods. (C) Comparison of the residual values between the random forest and SVM-RFE methods; the red dots represent the root mean square of the residuals. (D) Selection of the optimal parameter (λ) for LASSO regression through cross-validation. (E) Gene selection using LASSO regression. (F) The hub genes were selected as the intersection of genes identified by the random forest and LASSO algorithms. * RF, SVM-RFE, and LASSO analyses were performed using the TCGA-KIRC cohort. [file Image1.pdf]

**A**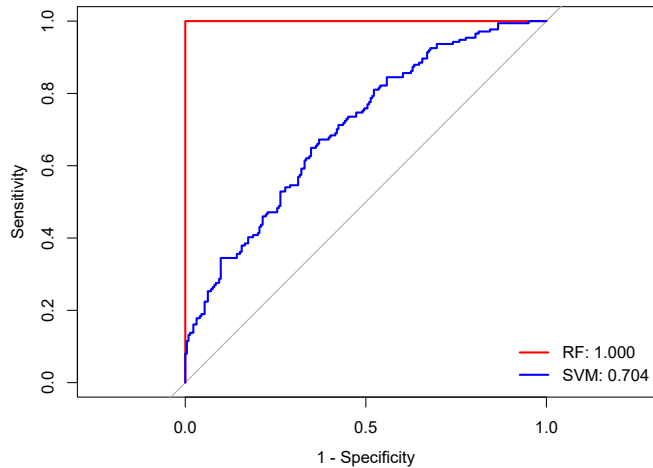**B**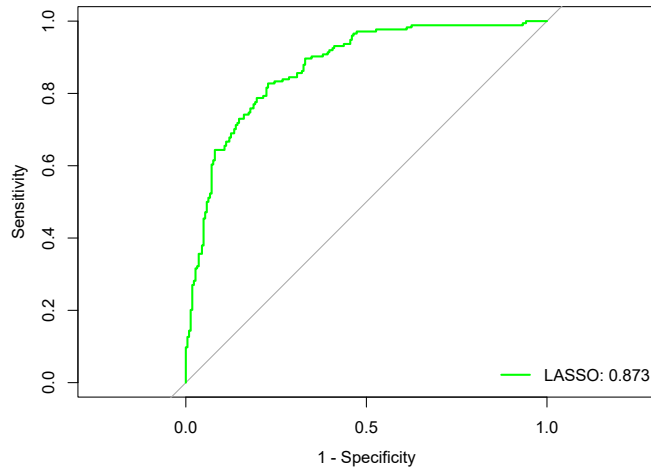

Supplement: Supplementary Figure 2 — (A) Comparison of the receiver operating characteristic (ROC) curves between the random forest and SVM methods in TCGA-KIRC cohort. (B) The ROC curve of LASSO method in TCGA-KIRC cohort. [file Image2.pdf]

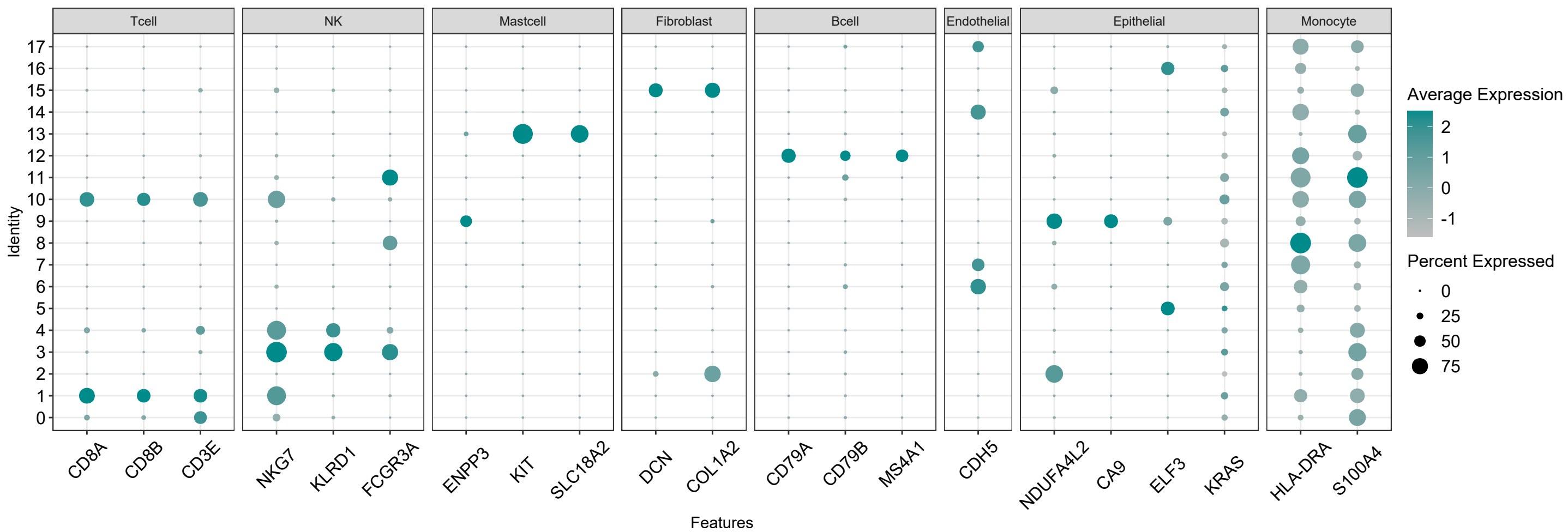

Supplement: Supplementary Figure 3 — Bubble plot depicting marker gene expression levels in each cell type. [file Image3.pdf]
