# Supplementary material for: Integrating machine learning and single-cell sequencing to identify shared biomarkers in type 1 diabetes mellitus and clear cell renal cell carcinoma
Source: Front Oncol. 2025 Mar 3;15:1543806. doi: 10.3389/fonc.2025.1543806 (PMC11911197; doi:10.3389/fonc.2025.1543806)
Supplement: Supplementary file 4 [file Table1.docx]

**Supplementary Table 1: Data source and sample information.**

| **Disease** | **Dataset** | **Data type** | **Sample information** |
| --- | --- | --- | --- |
| ccRCC | TCGA-KIRC | RNA-seq | 29 normal samples, 400 ccRCC samples |
|  | GSE53757 | Micro array (GPL570) | 72 normal samples, 72 ccRCC samples |
|  | GSE210042 | Single cell RNA-seq (GPL20301) | 7 ccRCC samples |
| T1DM | GSE55098 | Micro array (GPL570) | 10 normal PBMC samples, 12 T1DM PBMC samples |
|  | GSE9006 | Micro array (GPL97) | 24 normal PBMC samples, 43 T1DM PBMC samples |
