# Supplementary material for: Integrating machine learning and single-cell sequencing to identify shared biomarkers in type 1 diabetes mellitus and clear cell renal cell carcinoma
Source: Front Oncol. 2025 Mar 3;15:1543806. doi: 10.3389/fonc.2025.1543806 (PMC11911197; doi:10.3389/fonc.2025.1543806)
Supplement: Supplementary file 5 [file Table2.docx]

| Gene | Primer sequence | TM |
| --- | --- | --- |
| GAPDH | F: ACAGTTGCCATGTAGACC  R: TTTTTGGTTGAGCACAGG | 54.0  52.0 |
| KIF21A | F: ATACTGTGGGCGTTACAACAC  R: GTCCGGGATAAAGCACCCAAC | 60.3  63.0 |
| PIGH | F: GAGGATGAGCGGAGCTTTTC  R: GGCAGGACGGGGAGTAGTA | 60.5  62.1 |
| RPS6KA2 | F: GAAGAAGGCGTCGTGAAGGAG  R: CCGAACTTTTAGGGTGGCTTT | 62.7  60.5 |

**Supplementary Table 2: The mRNA-specific primer sequences.**
