# Supplementary material for: Integrating machine learning and single-cell sequencing to identify shared biomarkers in type 1 diabetes mellitus and clear cell renal cell carcinoma
Source: Front Oncol. 2025 Mar 3;15:1543806. doi: 10.3389/fonc.2025.1543806 (PMC11911197; doi:10.3389/fonc.2025.1543806)
Supplement: Supplementary file 7 [file Table4.docx]

**Supplementary Table 4: Patient information of the TCGA-KIRC cohort.**

| **Sample_id** | **Gender** | **Race** | **Age** | **Status** | **Pathologic_stage** | **T** | **N** | **M** |
| --- | --- | --- | --- | --- | --- | --- | --- | --- |
| TCGA-3Z-A93Z | MALE | BLACK OR AFRICAN AMERICAN | 69 | Alive | Stage I | T1a | N0 | M0 |
| TCGA-6D-AA2E | FEMALE | BLACK OR AFRICAN AMERICAN | 68 | Alive | Stage I | T1b | NX | MX |
| TCGA-A3-3306 | MALE | WHITE | 67 | Alive | Stage I | T1b | N0 | M0 |
| TCGA-A3-3307 | MALE | NA | 66 | Alive | Stage III | T3b | N0 | M0 |
| TCGA-A3-3308 | FEMALE | WHITE | 77 | Alive | Stage III | T3b | N0 | M0 |
| TCGA-A3-3311 | MALE | NA | 57 | Dead | Stage I | T1 | NX | M0 |
| TCGA-A3-3316 | MALE | WHITE | 57 | Alive | Stage II | T2 | NX | M0 |
| TCGA-A3-3317 | MALE | NA | 67 | Alive | Stage II | T2 | N0 | M0 |
| TCGA-A3-3319 | MALE | WHITE | 70 | Alive | Stage I | T1b | NX | M0 |
| TCGA-A3-3320 | FEMALE | WHITE | 52 | Alive | Stage I | T1b | NX | M0 |
| TCGA-A3-3322 | MALE | WHITE | 51 | Alive | Stage I | T1a | NX | M0 |
| TCGA-A3-3323 | MALE | WHITE | 53 | Alive | Stage I | T1b | NX | M0 |
| TCGA-A3-3324 | MALE | WHITE | 51 | Alive | Stage I | T1b | NX | M0 |
| TCGA-A3-3325 | MALE | WHITE | 52 | Alive | Stage I | T1a | NX | M0 |
| TCGA-A3-3326 | MALE | WHITE | 47 | Alive | Stage I | T1a | NX | M0 |
| TCGA-A3-3328 | MALE | WHITE | 79 | Alive | Stage I | T1b | N0 | M0 |
| TCGA-A3-3329 | MALE | WHITE | 75 | Alive | Stage I | T1b | N0 | M0 |
| TCGA-A3-3331 | FEMALE | WHITE | 86 | Alive | Stage I | T1 | N0 | M0 |
| TCGA-A3-3335 | MALE | WHITE | 41 | Alive | Stage II | T2a | N0 | M0 |
| TCGA-A3-3343 | MALE | WHITE | 79 | Alive | Stage II | T2 | N0 | M0 |
| TCGA-A3-3346 | MALE | WHITE | 68 | Dead | Stage I | T1b | NX | M0 |
| TCGA-A3-3347 | FEMALE | WHITE | 76 | Alive | Stage III | T1b | N1 | M0 |
| TCGA-A3-3349 | FEMALE | WHITE | 34 | Alive | Stage I | T1b | N0 | M0 |
| TCGA-A3-3351 | MALE | WHITE | 42 | Alive | Stage II | T2a | N0 | M0 |
| TCGA-A3-3357 | MALE | WHITE | 62 | Alive | Stage II | T2 | N0 | M0 |
| TCGA-A3-3358 | FEMALE | WHITE | 57 | Alive | Stage I | T1a | N0 | M0 |
| TCGA-A3-3359 | FEMALE | WHITE | 82 | Alive | Stage I | T1a | N0 | M0 |
| TCGA-A3-3362 | FEMALE | WHITE | 60 | Alive | Stage I | T1a | N0 | M0 |
| TCGA-A3-3363 | MALE | ASIAN | 50 | Alive | Stage II | T2 | N0 | M0 |
| TCGA-A3-3365 | MALE | WHITE | 46 | Alive | Stage I | T1a | NX | M0 |
| TCGA-A3-3367 | MALE | WHITE | 72 | Alive | Stage I | T1b | N0 | M0 |
| TCGA-A3-3370 | FEMALE | WHITE | 48 | Alive | Stage I | T1b | N0 | M0 |
| TCGA-A3-3372 | MALE | WHITE | 64 | Alive | Stage III | T3 | NX | M0 |
| TCGA-A3-3373 | FEMALE | WHITE | 54 | Alive | Stage I | T1b | N0 | M0 |
| TCGA-A3-3374 | FEMALE | BLACK OR AFRICAN AMERICAN | 51 | Alive | Stage I | T1b | N0 | M0 |
| TCGA-A3-3376 | MALE | BLACK OR AFRICAN AMERICAN | 51 | Alive | Stage I | T1a | N0 | M0 |
| TCGA-A3-3378 | MALE | WHITE | 60 | Alive | Stage I | T1 | N0 | M0 |
| TCGA-A3-3380 | MALE | NA | 54 | Alive | Stage I | T1 | N0 | M0 |
| TCGA-A3-3382 | MALE | WHITE | 69 | Alive | Stage I | T1b | NX | M0 |
| TCGA-A3-3383 | MALE | WHITE | 52 | Alive | Stage I | T1 | NX | M0 |
| TCGA-A3-3385 | FEMALE | WHITE | 46 | Alive | Stage I | T1a | N0 | M0 |
| TCGA-A3-A6NI | MALE | BLACK OR AFRICAN AMERICAN | 47 | Alive | Stage I | T1a | NX | M0 |
| TCGA-A3-A6NJ | FEMALE | BLACK OR AFRICAN AMERICAN | 57 | Alive | Stage I | T1a | NX | M0 |
| TCGA-A3-A6NL | FEMALE | BLACK OR AFRICAN AMERICAN | 49 | Alive | Stage I | T1b | NX | M0 |
| TCGA-A3-A6NN | MALE | BLACK OR AFRICAN AMERICAN | 78 | Alive | Stage I | T1a | NX | M0 |
| TCGA-A3-A8CQ | FEMALE | BLACK OR AFRICAN AMERICAN | 59 | Alive | Stage I | T1a | NX | M0 |
| TCGA-A3-A8OU | FEMALE | BLACK OR AFRICAN AMERICAN | 74 | Alive | Stage I | T1a | NX | M0 |
| TCGA-A3-A8OV | MALE | BLACK OR AFRICAN AMERICAN | 75 | Alive | Stage I | T1a | NX | M0 |
| TCGA-A3-A8OW | MALE | BLACK OR AFRICAN AMERICAN | 37 | Alive | Stage III | T1a | NX | M0 |
| TCGA-A3-A8OX | FEMALE | BLACK OR AFRICAN AMERICAN | 65 | Alive | Stage I | T1a | NX | M0 |
| TCGA-AK-3425 | MALE | WHITE | 68 | Alive | Stage I | T1 | N0 | M0 |
| TCGA-AK-3427 | MALE | WHITE | 65 | Alive | Stage I | T1a | N0 | M0 |
| TCGA-AK-3428 | MALE | WHITE | 62 | Alive | Stage III | T3b | N0 | M0 |
| TCGA-AK-3429 | FEMALE | WHITE | 54 | Alive | Stage II | T2 | N0 | M0 |
| TCGA-AK-3431 | FEMALE | WHITE | 62 | Alive | Stage II | T2 | NX | M0 |
| TCGA-AK-3433 | FEMALE | WHITE | 48 | Alive | Stage II | T2 | N0 | M0 |
| TCGA-AK-3434 | MALE | WHITE | 72 | Alive | Stage I | T1b | NX | M0 |
| TCGA-AK-3436 | MALE | WHITE | 40 | Alive | Stage IV | T2 | N0 | M1 |
| TCGA-AK-3440 | MALE | WHITE | 58 | Alive | Stage I | T1a | NX | M0 |
| TCGA-AK-3443 | MALE | WHITE | 45 | Alive | Stage II | T2 | N0 | M0 |
| TCGA-AK-3445 | MALE | WHITE | 69 | Alive | Stage III | T3a | NX | M0 |
| TCGA-AK-3447 | MALE | WHITE | 83 | Alive | Stage II | T2 | NX | M0 |
| TCGA-AK-3450 | FEMALE | WHITE | 85 | Alive | Stage I | T1a | N0 | M0 |
| TCGA-AK-3451 | MALE | WHITE | 48 | Alive | Stage II | T2 | N0 | M0 |
| TCGA-AK-3453 | FEMALE | WHITE | 58 | Alive | Stage II | T2 | NX | M0 |
| TCGA-AK-3454 | MALE | WHITE | 84 | Alive | Stage I | T1b | NX | M0 |
| TCGA-AK-3456 | MALE | BLACK OR AFRICAN AMERICAN | 48 | Alive | Stage II | T2 | N0 | M0 |
| TCGA-AK-3458 | MALE | WHITE | 48 | Alive | Stage I | T1b | NX | M0 |
| TCGA-AK-3460 | MALE | WHITE | 58 | Alive | Stage I | T1a | NX | M0 |
| TCGA-AK-3461 | MALE | WHITE | 72 | Alive | Stage I | T1a | NX | M0 |
| TCGA-AK-3465 | FEMALE | BLACK OR AFRICAN AMERICAN | 71 | Alive | Stage I | T1b | NX | M0 |
| TCGA-AS-3777 | MALE | WHITE | 63 | Alive | Stage I | T1a | NX | M0 |
| TCGA-AS-3778 | MALE | WHITE | 35 | Alive | Stage I | T1a | NX | M0 |
| TCGA-B0-4691 | MALE | WHITE | 55 | Dead | Stage IV | T2 | N0 | M1 |
| TCGA-B0-4694 | MALE | WHITE | 72 | Dead | Stage III | T3b | NX | M0 |
| TCGA-B0-4696 | MALE | WHITE | 58 | Dead | Stage III | T3a | N0 | M0 |
| TCGA-B0-4698 | MALE | WHITE | 75 | Dead | Stage IV | T4 | NX | M0 |
| TCGA-B0-4699 | MALE | WHITE | 74 | Dead | Stage IV | T4 | N0 | M1 |
| TCGA-B0-4706 | MALE | WHITE | 61 | Dead | Stage III | T3a | NX | M0 |
| TCGA-B0-4710 | FEMALE | WHITE | 75 | Alive | Stage III | T3a | N0 | M0 |
| TCGA-B0-4712 | MALE | WHITE | 76 | Dead | Stage IV | T3a | NX | M1 |
| TCGA-B0-4718 | MALE | WHITE | 57 | Alive | Stage III | T3a | NX | M0 |
| TCGA-B0-4811 | MALE | WHITE | 48 | Dead | Stage III | T3a | N0 | M0 |
| TCGA-B0-4813 | MALE | WHITE | 68 | Dead | Stage III | T3b | NX | M0 |
| TCGA-B0-4819 | FEMALE | WHITE | 60 | Dead | Stage IV | T3b | NX | M1 |
| TCGA-B0-4823 | MALE | WHITE | 88 | Dead | Stage I | T1a | N0 | M0 |
| TCGA-B0-4833 | FEMALE | WHITE | 82 | Dead | Stage I | T1b | N0 | M0 |
| TCGA-B0-4834 | MALE | WHITE | 49 | Dead | Stage I | T1a | N0 | M0 |
| TCGA-B0-4836 | MALE | WHITE | 61 | Dead | Stage IV | T3b | NX | M1 |
| TCGA-B0-4838 | FEMALE | WHITE | 69 | Dead | Stage I | T1b | N0 | M0 |
| TCGA-B0-4839 | FEMALE | WHITE | 80 | Dead | Stage I | T1b | N0 | M0 |
| TCGA-B0-4841 | MALE | WHITE | 63 | Dead | Stage IV | T2 | NX | M1 |
| TCGA-B0-4843 | MALE | WHITE | 57 | Dead | Stage III | T3a | N0 | M0 |
| TCGA-B0-4844 | MALE | WHITE | 60 | Dead | Stage IV | T3a | NX | M1 |
| TCGA-B0-4846 | MALE | WHITE | 52 | Dead | Stage IV | T3a | N0 | M1 |
| TCGA-B0-4848 | MALE | WHITE | 54 | Dead | Stage III | T3b | NX | M0 |
| TCGA-B0-4849 | MALE | WHITE | 51 | Dead | Stage III | T3a | NX | M0 |
| TCGA-B0-5075 | FEMALE | WHITE | 77 | Dead | Stage III | T3a | N0 | M0 |
| TCGA-B0-5092 | FEMALE | WHITE | 53 | Dead | Stage IV | T1a | N0 | M1 |
| TCGA-B0-5094 | MALE | WHITE | 62 | Dead | Stage IV | T3b | N0 | M1 |
| TCGA-B0-5097 | FEMALE | WHITE | 59 | Alive | Stage III | T3b | N0 | M0 |
| TCGA-B0-5098 | FEMALE | BLACK OR AFRICAN AMERICAN | 53 | Dead | Stage I | T1 | NX | M0 |
| TCGA-B0-5099 | FEMALE | WHITE | 88 | Dead | Stage III | T3b | NX | M0 |
| TCGA-B0-5100 | MALE | BLACK OR AFRICAN AMERICAN | 72 | Dead | Stage III | T3a | NX | M0 |
| TCGA-B0-5108 | MALE | WHITE | 54 | Alive | Stage III | T3a | N0 | M0 |
| TCGA-B0-5109 | MALE | WHITE | 69 | Dead | Stage III | T3b | N1 | M0 |
| TCGA-B0-5110 | FEMALE | WHITE | 71 | Alive | Stage I | T1a | N0 | M0 |
| TCGA-B0-5113 | FEMALE | WHITE | 69 | Alive | Stage III | T3a | N0 | M0 |
| TCGA-B0-5115 | MALE | WHITE | 43 | Alive | Stage IV | T2 | N0 | M1 |
| TCGA-B0-5116 | MALE | WHITE | 52 | Alive | Stage III | T3b | N0 | M0 |
| TCGA-B0-5117 | MALE | WHITE | 40 | Alive | Stage I | T1b | NX | M0 |
| TCGA-B0-5119 | FEMALE | WHITE | 61 | Alive | Stage I | T1b | N0 | M0 |
| TCGA-B0-5120 | FEMALE | WHITE | 72 | Alive | Stage I | T1a | N0 | M0 |
| TCGA-B0-5121 | MALE | WHITE | 56 | Alive | Stage I | T1b | N0 | M0 |
| TCGA-B0-5399 | MALE | WHITE | 46 | Alive | Stage I | T1b | N0 | M0 |
| TCGA-B0-5400 | FEMALE | WHITE | 59 | Alive | Stage III | T3b | N0 | M0 |
| TCGA-B0-5402 | MALE | WHITE | 64 | Alive | Stage IV | T4 | NX | M0 |
| TCGA-B0-5692 | FEMALE | WHITE | 66 | Alive | Stage III | T3b | N0 | M0 |
| TCGA-B0-5693 | FEMALE | WHITE | 47 | Alive | Stage I | T1b | NX | M0 |
| TCGA-B0-5695 | FEMALE | WHITE | 61 | Alive | Stage I | T1b | N0 | M0 |
| TCGA-B0-5697 | MALE | WHITE | 50 | Alive | Stage I | T1a | N0 | M0 |
| TCGA-B0-5698 | MALE | BLACK OR AFRICAN AMERICAN | 77 | Alive | Stage I | T1b | N0 | M0 |
| TCGA-B0-5700 | MALE | WHITE | 77 | Alive | Stage I | T1a | N0 | M0 |
| TCGA-B0-5702 | MALE | WHITE | 71 | Alive | Stage I | T1b | N0 | M0 |
| TCGA-B0-5705 | FEMALE | WHITE | 65 | Alive | Stage I | T1 | N0 | M0 |
| TCGA-B0-5706 | MALE | WHITE | 45 | Alive | Stage II | T2 | N0 | M0 |
| TCGA-B0-5707 | FEMALE | WHITE | 39 | Alive | Stage I | T1a | N0 | M0 |
| TCGA-B0-5709 | FEMALE | WHITE | 62 | Alive | Stage III | T3a | NX | M0 |
| TCGA-B0-5710 | MALE | WHITE | 57 | Alive | Stage I | T1b | N0 | M0 |
| TCGA-B0-5711 | MALE | WHITE | 50 | Alive | Stage III | T3b | NX | M0 |
| TCGA-B0-5713 | FEMALE | WHITE | 75 | Alive | Stage III | T3b | N0 | M0 |
| TCGA-B0-5812 | MALE | WHITE | 53 | Alive | Stage I | T1b | NX | M0 |
| TCGA-B2-3923 | MALE | WHITE | 59 | Alive | Stage II | T2 | NX | M0 |
| TCGA-B2-3924 | MALE | WHITE | 73 | Alive | Stage I | T1b | NX | M0 |
| TCGA-B2-4098 | FEMALE | BLACK OR AFRICAN AMERICAN | 72 | Dead | Stage I | T1b | NX | M0 |
| TCGA-B2-4099 | MALE | WHITE | 83 | Alive | Stage I | T1a | NX | M0 |
| TCGA-B2-4101 | MALE | WHITE | 52 | Alive | Stage II | T2a | NX | M0 |
| TCGA-B2-4102 | MALE | WHITE | 61 | Alive | Stage I | T1b | NX | M0 |
| TCGA-B2-5633 | MALE | WHITE | 56 | Alive | Stage I | T1b | N0 | M0 |
| TCGA-B2-5635 | MALE | WHITE | 74 | Alive | Stage I | T1a | NX | M0 |
| TCGA-B2-5639 | MALE | WHITE | 46 | Alive | Stage IV | T3 | NX | M1 |
| TCGA-B2-A4SR | MALE | BLACK OR AFRICAN AMERICAN | 61 | Alive | Stage II |  | NX | M0 |
| TCGA-B4-5377 | FEMALE | WHITE | 68 | Alive | Stage IV | T3 | N0 | M1 |
| TCGA-B4-5378 | MALE | WHITE | 62 | Alive | Stage I | T1 | N0 | M0 |
| TCGA-B4-5832 | MALE | WHITE | 65 | Alive | Stage III | T3b | N0 | M0 |
| TCGA-B4-5834 | MALE | WHITE | 59 | Alive | Stage I | T1 | N0 | M0 |
| TCGA-B4-5835 | FEMALE | WHITE | 64 | Alive | Stage I | T1 | N0 | M0 |
| TCGA-B4-5836 | FEMALE | WHITE | 61 | Alive | Stage I | T1b | N0 | M0 |
| TCGA-B4-5838 | MALE | WHITE | 52 | Alive |  | T3 | N1 | M0 |
| TCGA-B4-5843 | MALE | WHITE | 45 | Alive | Stage I | T1 | N0 | M0 |
| TCGA-B4-5844 | FEMALE | WHITE | 61 | Alive | Stage II | T2 | N0 | M0 |
| TCGA-B8-4143 | FEMALE | WHITE | 66 | Dead | Stage IV | T3a | N0 | M1 |
| TCGA-B8-4146 | FEMALE | WHITE | 41 | Alive | Stage I | T1b | NX | M0 |
| TCGA-B8-4148 | FEMALE | WHITE | 63 | Alive | Stage I | T1a | N0 | M0 |
| TCGA-B8-4151 | FEMALE | WHITE | 51 | Alive | Stage III | T3a | N0 | M0 |
| TCGA-B8-4153 | MALE | WHITE | 74 | Alive | Stage III | T3a | NX | M0 |
| TCGA-B8-4154 | FEMALE | WHITE | 73 | Alive | Stage I | T1a | N0 | M0 |
| TCGA-B8-4619 | MALE | WHITE | 58 | Alive | Stage I | T1a | N0 | M0 |
| TCGA-B8-4621 | MALE | BLACK OR AFRICAN AMERICAN | 63 | Alive | Stage I | T1b | N0 | M0 |
| TCGA-B8-5158 | MALE | WHITE | 56 | Alive | Stage III | T3a | N1 | M0 |
| TCGA-B8-5159 | FEMALE | WHITE | 61 | Alive | Stage I | T1a | N0 | M0 |
| TCGA-B8-5162 | MALE | WHITE | 62 | Alive | Stage II | T2a | NX | M0 |
| TCGA-B8-5163 | FEMALE | WHITE | 63 | Alive | Stage III | T3a | N0 | M0 |
| TCGA-B8-5164 | MALE | WHITE | 65 | Alive | Stage III | T3a | N0 | M0 |
| TCGA-B8-5165 | MALE | WHITE | 43 | Alive | Stage I | T1a | N0 | M0 |
| TCGA-B8-5545 | MALE | BLACK OR AFRICAN AMERICAN | 42 | Alive | Stage I | T1a | N0 | M0 |
| TCGA-B8-5546 | FEMALE | BLACK OR AFRICAN AMERICAN | 38 | Alive | Stage I | T1b | N0 | M0 |
| TCGA-B8-5549 | MALE | WHITE | 53 | Alive | Stage I | T1b | N0 | M0 |
| TCGA-B8-5550 | MALE | WHITE | 71 | Alive | Stage III | T3a | N0 | M0 |
| TCGA-B8-5551 | FEMALE | BLACK OR AFRICAN AMERICAN | 65 | Alive | Stage I | T1b | N0 | M0 |
| TCGA-B8-5553 | FEMALE | WHITE | 67 | Alive | Stage I | T1b | N0 | M0 |
| TCGA-B8-A54D | MALE | BLACK OR AFRICAN AMERICAN | 69 | Alive | Stage III | T2a | NX | M0 |
| TCGA-B8-A54E | FEMALE | BLACK OR AFRICAN AMERICAN | 62 | Alive | Stage I | T0 | NX | M0 |
| TCGA-B8-A54F | FEMALE | BLACK OR AFRICAN AMERICAN | 49 | Alive | Stage I | T1a | NX | M0 |
| TCGA-B8-A54G | MALE | BLACK OR AFRICAN AMERICAN | 50 | Alive | Stage I | T1a | NX | M0 |
| TCGA-B8-A54H | FEMALE | BLACK OR AFRICAN AMERICAN | 69 | Alive | Stage II | T2a | NX | M0 |
| TCGA-B8-A54I | MALE | BLACK OR AFRICAN AMERICAN | 48 | Alive | Stage I | T0 | NX | M0 |
| TCGA-B8-A54J | MALE | BLACK OR AFRICAN AMERICAN | 60 | Alive | Stage II | T2a | NX | M0 |
| TCGA-B8-A54K | MALE | BLACK OR AFRICAN AMERICAN | 61 | Alive | Stage I | T1a | NX | M0 |
| TCGA-B8-A7U6 | FEMALE | BLACK OR AFRICAN AMERICAN | 54 | Alive | Stage I | T1a | NX | M0 |
| TCGA-B8-A8YJ | FEMALE | BLACK OR AFRICAN AMERICAN | 60 | Alive | Stage I | T0 | NX | M0 |
| TCGA-BP-4158 | MALE | WHITE | 69 | Alive | Stage I | T1b | N0 | M0 |
| TCGA-BP-4160 | MALE | WHITE | 67 | Alive | Stage III | T3a | N0 | M0 |
| TCGA-BP-4161 | MALE | WHITE | 74 | Alive | Stage I | T1b | NX | M0 |
| TCGA-BP-4162 | FEMALE | WHITE | 65 | Alive | Stage I | T1b | N0 | M0 |
| TCGA-BP-4163 | FEMALE | WHITE | 60 | Alive | Stage III | T3a | N0 | M0 |
| TCGA-BP-4164 | FEMALE | WHITE | 51 | Dead | Stage III | T3a | NX | M0 |
| TCGA-BP-4165 | FEMALE | WHITE | 64 | Alive | Stage I | T1b | N0 | M0 |
| TCGA-BP-4166 | MALE | WHITE | 69 | Alive | Stage III | T3a | N0 | M0 |
| TCGA-BP-4167 | MALE | WHITE | 59 | Alive | Stage III | T3a | NX | M0 |
| TCGA-BP-4169 | FEMALE | WHITE | 76 | Dead | Stage II | T2 | N0 | M0 |
| TCGA-BP-4170 | FEMALE | WHITE | 72 | Dead | Stage I | T1b | N0 | M0 |
| TCGA-BP-4173 | MALE | WHITE | 47 | Alive | Stage II | T2 | N0 | M0 |
| TCGA-BP-4174 | MALE | WHITE | 49 | Alive | Stage II | T2 | N0 | M0 |
| TCGA-BP-4176 | MALE | WHITE | 64 | Alive | Stage I | T1b | NX | M0 |
| TCGA-BP-4177 | MALE | WHITE | 65 | Alive | Stage I | T1a | NX | M0 |
| TCGA-BP-4325 | FEMALE | WHITE | 64 | Alive | Stage I | T1b | N0 | M0 |
| TCGA-BP-4327 | FEMALE | WHITE | 75 | Dead | Stage II | T2 | N0 | M0 |
| TCGA-BP-4330 | FEMALE | BLACK OR AFRICAN AMERICAN | 60 | Alive | Stage III | T3a | N0 | M0 |
| TCGA-BP-4331 | MALE | WHITE | 52 | Dead | Stage I | T1a | N0 | M0 |
| TCGA-BP-4332 | MALE | WHITE | 36 | Alive | Stage III | T3a | N0 | M0 |
| TCGA-BP-4338 | MALE | WHITE | 43 | Alive | Stage I | T1b | N0 | M0 |
| TCGA-BP-4344 | FEMALE | WHITE | 75 | Alive | Stage I | T1a | NX | M0 |
| TCGA-BP-4345 | MALE | WHITE | 62 | Alive | Stage III | T3b | N0 | M0 |
| TCGA-BP-4346 | MALE | WHITE | 57 | Dead | Stage III | T3b | N0 | M0 |
| TCGA-BP-4347 | MALE | WHITE | 74 | Alive | Stage III | T3b | NX | M0 |
| TCGA-BP-4349 | FEMALE | WHITE | 68 | Alive | Stage I | T1a | NX | M0 |
| TCGA-BP-4351 | FEMALE | WHITE | 51 | Alive | Stage III | T3a | N0 | M0 |
| TCGA-BP-4353 | MALE | WHITE | 61 | Dead | Stage I | T1 | N0 | M0 |
| TCGA-BP-4756 | FEMALE | ASIAN | 62 | Alive | Stage I | T1b | N0 | M0 |
| TCGA-BP-4758 | MALE | WHITE | 40 | Alive | Stage I | T1a | NX | M0 |
| TCGA-BP-4759 | MALE | WHITE | 50 | Alive | Stage I | T1a | NX | M0 |
| TCGA-BP-4760 | MALE | BLACK OR AFRICAN AMERICAN | 69 | Alive | Stage I | T1a | NX | M0 |
| TCGA-BP-4761 | MALE | WHITE | 57 | Alive | Stage III | T3a | N1 | M0 |
| TCGA-BP-4762 | MALE | WHITE | 42 | Dead | Stage I | T1a | NX | M0 |
| TCGA-BP-4765 | MALE | WHITE | 43 | Alive | Stage I | T1a | NX | M0 |
| TCGA-BP-4766 | FEMALE | WHITE | 43 | Alive | Stage I | T1a | NX | M0 |
| TCGA-BP-4768 | FEMALE | WHITE | 72 | Alive | Stage I | T1a | N0 | M0 |
| TCGA-BP-4769 | MALE | WHITE | 63 | Alive | Stage I | T1a | NX | M0 |
| TCGA-BP-4771 | MALE | WHITE | 62 | Dead | Stage IV | T3a | N0 | M1 |
| TCGA-BP-4774 | FEMALE | WHITE | 57 | Alive | Stage I | T1a | NX | M0 |
| TCGA-BP-4775 | FEMALE | WHITE | 55 | Alive | Stage I | T1a | NX | M0 |
| TCGA-BP-4776 | MALE | WHITE | 52 | Alive | Stage I | T1a | NX | M0 |
| TCGA-BP-4777 | MALE | WHITE | 46 | Alive | Stage I | T1a | NX | M0 |
| TCGA-BP-4781 | MALE | WHITE | 78 | Alive | Stage I | T1a | NX | M0 |
| TCGA-BP-4782 | FEMALE | WHITE | 55 | Alive | Stage I | T1a | NX | M0 |
| TCGA-BP-4784 | FEMALE | WHITE | 67 | Alive | Stage I | T1a | NX | M0 |
| TCGA-BP-4789 | MALE | WHITE | 48 | Alive | Stage I | T1a | NX | M0 |
| TCGA-BP-4795 | FEMALE | WHITE | 74 | Alive | Stage I | T1a | N0 | M0 |
| TCGA-BP-4797 | MALE | WHITE | 34 | Alive | Stage III | T3b | N0 | M0 |
| TCGA-BP-4799 | MALE | WHITE | 70 | Dead | Stage III | T3b | N0 | M0 |
| TCGA-BP-4801 | MALE | WHITE | 57 | Alive | Stage I | T1a | NX | M0 |
| TCGA-BP-4803 | MALE | WHITE | 79 | Alive | Stage III | T3a | NX | M0 |
| TCGA-BP-4804 | MALE | WHITE | 59 | Alive | Stage I | T1b | NX | M0 |
| TCGA-BP-4807 | MALE | WHITE | 42 | Alive | Stage I | T1a | NX | M0 |
| TCGA-BP-4959 | MALE | WHITE | 49 | Alive | Stage I | T1b | NX | M0 |
| TCGA-BP-4960 | MALE | WHITE | 46 | Alive | Stage II | T2 | N0 | M0 |
| TCGA-BP-4961 | MALE | WHITE | 47 | Alive | Stage I | T1a | NX | M0 |
| TCGA-BP-4962 | MALE | WHITE | 58 | Alive | Stage II | T2 | NX | M0 |
| TCGA-BP-4963 | MALE | WHITE | 63 | Alive | Stage I | T1b | NX | M0 |
| TCGA-BP-4964 | FEMALE | WHITE | 54 | Alive | Stage I | T1a | N0 | M0 |
| TCGA-BP-4965 | MALE | WHITE | 46 | Alive | Stage I | T1a | NX | M0 |
| TCGA-BP-4967 | MALE | WHITE | 76 | Alive | Stage III | T3a | N0 | M0 |
| TCGA-BP-4968 | MALE | WHITE | 40 | Alive | Stage I | T1b | N0 | M0 |
| TCGA-BP-4969 | FEMALE | WHITE | 63 | Alive | Stage I | T1a | NX | M0 |
| TCGA-BP-4970 | MALE | ASIAN | 44 | Alive | Stage III | T1a | N1 | M0 |
| TCGA-BP-4971 | MALE | WHITE | 40 | Alive | Stage III | T3a | N0 | M0 |
| TCGA-BP-4972 | FEMALE | WHITE | 43 | Alive | Stage III | T3a | NX | M0 |
| TCGA-BP-4973 | MALE | WHITE | 47 | Alive | Stage III | T3a | NX | M0 |
| TCGA-BP-4975 | MALE | WHITE | 40 | Alive | Stage I | T1b | NX | M0 |
| TCGA-BP-4976 | MALE | WHITE | 77 | Alive | Stage I | T1a | NX | M0 |
| TCGA-BP-4977 | MALE | WHITE | 57 | Alive | Stage I | T1b | NX | M0 |
| TCGA-BP-4981 | FEMALE | WHITE | 75 | Dead | Stage III | T3a | NX | M0 |
| TCGA-BP-4982 | MALE | WHITE | 42 | Alive | Stage I | T1b | NX | M0 |
| TCGA-BP-4983 | FEMALE | ASIAN | 67 | Alive | Stage III | T3a | NX | M0 |
| TCGA-BP-4986 | MALE | WHITE | 75 | Alive | Stage I | T1a | N0 | M0 |
| TCGA-BP-4987 | FEMALE | ASIAN | 41 | Alive | Stage I | T1b | NX | M0 |
| TCGA-BP-4989 | MALE | WHITE | 58 | Alive | Stage III | T3a | N0 | M0 |
| TCGA-BP-4991 | MALE | WHITE | 54 | Alive | Stage I | T1a | NX | M0 |
| TCGA-BP-4992 | MALE | WHITE | 66 | Alive | Stage I | T1b | NX | M0 |
| TCGA-BP-4993 | MALE | WHITE | 58 | Alive | Stage I | T1a | NX | M0 |
| TCGA-BP-4994 | MALE | WHITE | 54 | Alive | Stage I | T1a | NX | M0 |
| TCGA-BP-4995 | MALE | WHITE | 68 | Alive | Stage I | T1b | N0 | M0 |
| TCGA-BP-4998 | MALE | ASIAN | 49 | Alive | Stage I | T1a | NX | M0 |
| TCGA-BP-4999 | MALE | WHITE | 56 | Alive | Stage I | T1a | NX | M0 |
| TCGA-BP-5000 | MALE | WHITE | 40 | Alive | Stage I | T1b | NX | M0 |
| TCGA-BP-5001 | FEMALE | WHITE | 43 | Alive | Stage I | T1b | NX | M0 |
| TCGA-BP-5004 | MALE | WHITE | 53 | Alive | Stage I | T1a | NX | M0 |
| TCGA-BP-5006 | MALE | WHITE | 61 | Alive | Stage I | T1a | N0 | M0 |
| TCGA-BP-5007 | MALE | WHITE | 45 | Alive | Stage II | T2 | N0 | M0 |
| TCGA-BP-5008 | MALE | WHITE | 46 | Alive | Stage I | T1a | NX | M0 |
| TCGA-BP-5009 | MALE | WHITE | 52 | Dead | Stage I | T1b | NX | M0 |
| TCGA-BP-5168 | MALE | WHITE | 75 | Dead | Stage I | T1a | NX | M0 |
| TCGA-BP-5169 | MALE | WHITE | 70 | Alive | Stage I | T1b | N0 | M0 |
| TCGA-BP-5170 | MALE | WHITE | 55 | Alive | Stage I | T1a | NX | M0 |
| TCGA-BP-5174 | FEMALE | WHITE | 45 | Alive | Stage I | T1a | NX | M0 |
| TCGA-BP-5175 | MALE | WHITE | 60 | Alive | Stage I | T1a | NX | M0 |
| TCGA-BP-5176 | FEMALE | WHITE | 78 | Dead | Stage I | T1a | NX | M0 |
| TCGA-BP-5177 | FEMALE | WHITE | 46 | Alive | Stage I | T1a | NX | M0 |
| TCGA-BP-5178 | MALE | WHITE | 71 | Dead | Stage IV | T3a | NX | M1 |
| TCGA-BP-5180 | MALE | WHITE | 53 | Alive | Stage I | T1a | NX | M0 |
| TCGA-BP-5181 | FEMALE | WHITE | 58 | Alive | Stage I | T1b | NX | M0 |
| TCGA-BP-5182 | MALE | WHITE | 56 | Alive | Stage I | T1a | N0 | M0 |
| TCGA-BP-5183 | MALE | WHITE | 57 | Alive | Stage III | T3a | NX | M0 |
| TCGA-BP-5184 | MALE | WHITE | 54 | Alive | Stage I | T1a | NX | M0 |
| TCGA-BP-5185 | MALE | WHITE | 56 | Alive | Stage I | T1a | NX | M0 |
| TCGA-BP-5186 | FEMALE | WHITE | 50 | Alive | Stage I | T1a | N0 | M0 |
| TCGA-BP-5187 | MALE | WHITE | 54 | Alive | Stage I | T1a | NX | M0 |
| TCGA-BP-5190 | MALE | WHITE | 61 | Alive | Stage I | T1a | NX | M0 |
| TCGA-BP-5191 | MALE | WHITE | 79 | Alive | Stage III | T3a | N0 | M0 |
| TCGA-BP-5192 | MALE | WHITE | 59 | Alive | Stage I | T1a | NX | M0 |
| TCGA-BP-5194 | MALE | WHITE | 39 | Alive | Stage I | T1a | NX | M0 |
| TCGA-BP-5195 | MALE | WHITE | 75 | Alive | Stage I | T1a | NX | M0 |
| TCGA-BP-5196 | MALE | WHITE | 53 | Alive | Stage I | T1a | NX | M0 |
| TCGA-BP-5198 | MALE | WHITE | 72 | Alive | Stage III | T3b | N0 | M0 |
| TCGA-BP-5199 | MALE | WHITE | 58 | Alive | Stage II | T2 | N0 | M0 |
| TCGA-BP-5200 | MALE | WHITE | 44 | Alive | Stage II | T2 | NX | M0 |
| TCGA-BP-5201 | MALE | WHITE | 63 | Alive | Stage IV | T3b | N0 | M1 |
| TCGA-BP-5202 | MALE | WHITE | 75 | Alive | Stage III | T3a | NX | M0 |
| TCGA-CJ-4634 | FEMALE | WHITE | 60 | Alive | Stage I | T1b | NX | M0 |
| TCGA-CJ-4635 | MALE | WHITE | 48 | Alive | Stage I | T1b | NX | M0 |
| TCGA-CJ-4636 | MALE | WHITE | 51 | Alive | Stage III | T3a | N0 | M0 |
| TCGA-CJ-4639 | FEMALE | WHITE | 49 | Alive | Stage II | T2 | N0 | M0 |
| TCGA-CJ-4640 | MALE | WHITE | 49 | Alive | Stage III | T3a | N0 | M0 |
| TCGA-CJ-4642 | MALE | WHITE | 47 | Alive | Stage II | T2 | NX | M0 |
| TCGA-CJ-4643 | FEMALE | WHITE | 67 | Alive | Stage II | T2b | N0 | M0 |
| TCGA-CJ-4869 | MALE | WHITE | 49 | Alive | Stage III | T2 | N1 | M0 |
| TCGA-CJ-4870 | FEMALE | BLACK OR AFRICAN AMERICAN | 58 | Alive | Stage III | T3a | NX | M0 |
| TCGA-CJ-4871 | MALE | WHITE | 63 | Alive | Stage IV | T3a | NX | M1 |
| TCGA-CJ-4872 | MALE | WHITE | 51 | Alive | Stage I | T1b | N0 | M0 |
| TCGA-CJ-4873 | FEMALE | WHITE | 85 | Alive | Stage III | T3a | N0 | M0 |
| TCGA-CJ-4874 | FEMALE | WHITE | 73 | Alive | Stage I | T1b | N0 | M0 |
| TCGA-CJ-4875 | MALE | WHITE | 67 | Alive | Stage IV | T3a | NX | M1 |
| TCGA-CJ-4876 | MALE | WHITE | 57 | Alive | Stage II | T2b | N0 | M0 |
| TCGA-CJ-4878 | FEMALE | WHITE | 71 | Alive | Stage III | T3a | NX | M0 |
| TCGA-CJ-4881 | MALE | WHITE | 41 | Alive | Stage III | T3a | NX | M0 |
| TCGA-CJ-4882 | MALE | BLACK OR AFRICAN AMERICAN | 57 | Alive | Stage III | T3a | NX | M0 |
| TCGA-CJ-4884 | FEMALE | WHITE | 72 | Alive | Stage III | T3a | NX | M0 |
| TCGA-CJ-4885 | MALE | WHITE | 64 | Alive | Stage IV | T3a | NX | M1 |
| TCGA-CJ-4886 | FEMALE | WHITE | 42 | Alive | Stage I | T1a | NX | M0 |
| TCGA-CJ-4888 | MALE | WHITE | 59 | Dead | Stage IV | T3a | NX | M1 |
| TCGA-CJ-4889 | FEMALE | WHITE | 63 | Alive | Stage I | T1a | NX | M0 |
| TCGA-CJ-4890 | MALE | WHITE | 72 | Alive | Stage IV | T3a | N0 | M1 |
| TCGA-CJ-4891 | FEMALE | WHITE | 57 | Dead | Stage III | T3c | N0 | M0 |
| TCGA-CJ-4892 | FEMALE | WHITE | 65 | Alive | Stage I | T1b | N0 | M0 |
| TCGA-CJ-4893 | FEMALE | WHITE | 76 | Alive | Stage I | T1b | NX | M0 |
| TCGA-CJ-4894 | MALE | WHITE | 58 | Dead | Stage III | T3a | N0 | M0 |
| TCGA-CJ-4897 | FEMALE | WHITE | 79 | Alive | Stage III | T3a | NX | M0 |
| TCGA-CJ-4899 | MALE | ASIAN | 42 | Alive | Stage I | T1b | NX | M0 |
| TCGA-CJ-4901 | MALE | WHITE | 47 | Alive | Stage III | T3b | NX | M0 |
| TCGA-CJ-4902 | MALE | WHITE | 61 | Alive | Stage III | T3a | NX | M0 |
| TCGA-CJ-4903 | MALE | WHITE | 50 | Alive | Stage I | T1b | NX | M0 |
| TCGA-CJ-4904 | FEMALE | WHITE | 60 | Alive | Stage IV | T3a | N0 | M1 |
| TCGA-CJ-4905 | FEMALE | WHITE | 62 | Alive | Stage I | T1a | NX | M0 |
| TCGA-CJ-4907 | MALE | WHITE | 58 | Alive | Stage III | T3b | NX | M0 |
| TCGA-CJ-4908 | MALE | WHITE | 38 | Alive | Stage I | T1a | NX | M0 |
| TCGA-CJ-4912 | MALE | WHITE | 61 | Alive | Stage II | T2 | NX | M0 |
| TCGA-CJ-4916 | FEMALE | WHITE | 69 | Alive | Stage III | T3a | NX | M0 |
| TCGA-CJ-4920 | FEMALE | WHITE | 64 | Dead | Stage I | T1b | NX | M0 |
| TCGA-CJ-5671 | MALE | WHITE | 51 | Alive | Stage I | T1a | NX | M0 |
| TCGA-CJ-5675 | MALE | WHITE | 70 | Alive | Stage II | T2a | NX | M0 |
| TCGA-CJ-5676 | MALE | WHITE | 47 | Alive | Stage III | T3b | NX | M0 |
| TCGA-CJ-5682 | MALE | WHITE | 60 | Alive | Stage IV | T3a | NX | M1 |
| TCGA-CJ-5683 | MALE | WHITE | 78 | Alive | Stage I | T1b | NX | M0 |
| TCGA-CJ-5684 | MALE | WHITE | 61 | Alive | Stage III | T3a | NX | M0 |
| TCGA-CJ-5686 | FEMALE | WHITE | 59 | Alive | Stage I | T1b | NX | M0 |
| TCGA-CJ-6027 | MALE | WHITE | 77 | Alive | Stage I | T1a | NX | M0 |
| TCGA-CJ-6031 | MALE | WHITE | 54 | Alive | Stage I | T1b | NX | M0 |
| TCGA-CJ-6032 | FEMALE | WHITE | 63 | Alive | Stage II | T2 | NX | M0 |
| TCGA-CW-5580 | FEMALE | WHITE | 73 | Dead | Stage IV | T3a | NX | M1 |
| TCGA-CW-5583 | FEMALE | WHITE | 51 | Alive | Stage I | T1a | NX | M0 |
| TCGA-CW-5585 | MALE | WHITE | 51 | Alive | Stage IV | T3b | N0 | M1 |
| TCGA-CW-5587 | FEMALE | WHITE | 62 | Alive | Stage III | T3b | N0 | M0 |
| TCGA-CW-5588 | FEMALE | WHITE | 78 | Alive | Stage I | T1a | NX | M0 |
| TCGA-CW-5589 | MALE | WHITE | 52 | Alive | Stage I | T1a | NX | M0 |
| TCGA-CW-5591 | MALE | WHITE | 56 | Alive | Stage IV | T3a | N0 | M1 |
| TCGA-CW-6093 | MALE | WHITE | 73 | Alive | Stage I | T1a | NX | M0 |
| TCGA-CW-6097 | MALE | WHITE | 32 | Dead | Stage III | T3a | NX | M0 |
| TCGA-CZ-4853 | MALE | WHITE | 82 | Alive | Stage I | T1a | NX | M0 |
| TCGA-CZ-4856 | FEMALE | WHITE | 62 | Alive | Stage I | T1b | N0 | M0 |
| TCGA-CZ-4858 | MALE | WHITE | 39 | Alive | Stage II | T2 | NX | M0 |
| TCGA-CZ-4859 | FEMALE | WHITE | 59 | Alive | Stage I | T1 | N0 | M0 |
| TCGA-CZ-4862 | MALE | WHITE | 46 | Alive | Stage I | T1b | NX | M0 |
| TCGA-CZ-4863 | FEMALE | WHITE | 51 | Alive | Stage III | T3b | N0 | M0 |
| TCGA-CZ-4866 | FEMALE | WHITE | 79 | Alive | Stage I | T1 | NX | M0 |
| TCGA-CZ-5451 | MALE | WHITE | 74 | Alive | Stage II | T2 | N0 | M0 |
| TCGA-CZ-5455 | MALE | WHITE | 63 | Dead | Stage IV | T3b | NX | M1 |
| TCGA-CZ-5459 | MALE | WHITE | 63 | Alive | Stage III | T3b | NX | M0 |
| TCGA-CZ-5460 | MALE | WHITE | 55 | Alive | Stage IV | T3b | NX | M1 |
| TCGA-CZ-5461 | MALE | WHITE | 52 | Dead | Stage IV | T1b | NX | M1 |
| TCGA-CZ-5464 | MALE | WHITE | 69 | Alive | Stage IV | T3b | NX | M1 |
| TCGA-CZ-5465 | FEMALE | NA | 76 | Alive | Stage III | T3b | NX | M0 |
| TCGA-CZ-5466 | MALE | NA | 67 | Alive | Stage III | T3a | NX | M0 |
| TCGA-CZ-5468 | MALE | WHITE | 84 | Dead | Stage IV | T3b | NX | M1 |
| TCGA-CZ-5470 | FEMALE | NA | 72 | Alive | Stage II | T2 | N0 | M0 |
| TCGA-CZ-5982 | FEMALE | WHITE | 59 | Alive | Stage I | T1a | NX | M0 |
| TCGA-CZ-5989 | MALE | WHITE | 60 | Alive | Stage II | T2 | N0 | M0 |
| TCGA-DV-5565 | MALE | WHITE | 59 | Alive | Stage I | T1a | NX | M0 |
| TCGA-DV-5566 | FEMALE | WHITE | 67 | Alive | Stage I | T1a | NX | M0 |
| TCGA-DV-5567 | FEMALE | BLACK OR AFRICAN AMERICAN | 40 | Alive | Stage I | T1a | NX | M0 |
| TCGA-DV-5568 | MALE | WHITE | 26 | Alive | Stage I | T1a | NX | M0 |
| TCGA-DV-5569 | FEMALE | WHITE | 29 | Alive | Stage I | T1a | NX | M0 |
| TCGA-DV-5573 | MALE | WHITE | 41 | Alive | Stage I | T1a | NX | M0 |
| TCGA-DV-5574 | MALE | WHITE | 37 | Alive | Stage I | T1a | NX | M0 |
| TCGA-DV-5575 | FEMALE | WHITE | 52 | Alive | Stage I | T1a | NX | M0 |
| TCGA-DV-A4VZ | MALE | BLACK OR AFRICAN AMERICAN | 53 | Alive | Stage I | T0a | NX | MX |
| TCGA-DV-A4W0 | MALE | BLACK OR AFRICAN AMERICAN | 55 | Alive | Stage I | T1b | NX | M0 |
| TCGA-EU-5904 | FEMALE | WHITE | 47 | Alive | Stage I | T1 | NX | M0 |
| TCGA-EU-5905 | FEMALE | WHITE | 67 | Alive | Stage I | T1 | NX | M0 |
| TCGA-EU-5906 | MALE | WHITE | 55 | Alive | Stage I | T1b | NX | M0 |
| TCGA-EU-5907 | MALE | WHITE | 81 | Alive | Stage III | T3a | NX | M0 |
| TCGA-G6-A8L6 | MALE | BLACK OR AFRICAN AMERICAN | 55 | Alive | Stage IV | T1a | NX | M1 |
| TCGA-G6-A8L7 | FEMALE | BLACK OR AFRICAN AMERICAN | 81 | Alive | Stage I | T1b | N1 | MX |
| TCGA-G6-A8L8 | FEMALE | BLACK OR AFRICAN AMERICAN | 62 | Alive | Stage I | T1b | NX | MX |
| TCGA-GK-A6C7 | FEMALE | BLACK OR AFRICAN AMERICAN | 76 | Alive | Stage I | T1a | NX | MX |
| TCGA-MM-A563 | MALE | BLACK OR AFRICAN AMERICAN | 41 | Alive | Stage III | T2 | NX | MX |
| TCGA-MM-A564 | MALE | BLACK OR AFRICAN AMERICAN | 68 | Alive | Stage II | T2a | NX | MX |
| TCGA-MM-A84U | FEMALE | BLACK OR AFRICAN AMERICAN | 58 | Alive | Stage I | T1a | NX | MX |
| TCGA-MW-A4EC | FEMALE | BLACK OR AFRICAN AMERICAN | 72 | Alive | Stage I | T1a | NX | MX |
| TCGA-T7-A92I | FEMALE | BLACK OR AFRICAN AMERICAN | 47 | Alive | Stage I | T1a | NX | MX |
